# NPD1 Plus RvD1 Mediated Ischemic Stroke Penumbra Protection Increases Expression of Pro-homeostatic Microglial and Astrocyte Genes

**DOI:** 10.1007/s10571-023-01363-3

**Published:** 2023-06-04

**Authors:** Madigan M. Reid, Marie-Audrey I. Kautzmann, Gethein Andrew, Andre Obenaus, Pranab K. Mukherjee, Larissa Khoutorova, Jeff X. Ji, Cassia R. Roque, Reinaldo B. Oria, Bola F. Habeb, Ludmila Belayev, Nicolas G. Bazan

**Affiliations:** 1 Neuroscience Center of Excellence, School of Medicine, Louisiana State University Health Sciences Center, New Orleans, LA 70112 USA; 2 Department of Pediatrics, School of Medicine, University of California, Irvine, CA 92618 USA; 3 Laboratory of the Biology of Tissue Healing, Ontogeny and Nutrition, Department of Morphology and Institute of Biomedicine, School of Medicine, Federal University of Ceara, Fortaleza, Brazil; 4 Neuroscience Center of Excellence, School of Medicine, Louisiana State University Health New Orleans, Neuroscience Center of Excellence, 2020 Gravier Street, Suite D, New Orleans, LA 70112 USA; 5 Neuroscience Center of Excellence, School of Medicine, Louisiana State University Health New Orleans, 2020 Gravier St, Suite 9B16, Room 935A, New Orleans, LA 70112 USA

**Keywords:** Neuroprotection, Ischemic core, Lipid mediators, Apoptosis

## Abstract

Neuroprotection to attenuate or block the ischemic cascade and salvage neuronal damage has been extensively explored for treating ischemic stroke. However, despite increasing knowledge of the physiologic, mechanistic, and imaging characterizations of the ischemic penumbra, no effective neuroprotective therapy has been found. This study focuses on the neuroprotective bioactivity of docosanoid mediators: Neuroprotectin D1 (NPD1), Resolvin D1 (RvD1), and their combination in experimental stroke. Molecular targets of NPD1 and RvD1 are defined by following dose–response and therapeutic window. We demonstrated that treatment with NPD1, RvD1, and combination therapy provides high-grade neurobehavioral recovery and decreases ischemic core and penumbra volumes even when administered up to 6 h after stroke. The expression of the following genes was salient: (a) *Cd163,* an anti-inflammatory stroke-associated gene, was the most differentially expressed gene by NPD1+RvD1, displaying more than a 123-fold upregulation in the ipsilesional penumbra (Lisi et al., Neurosci Lett 645:106–112, 2017); (b) 100-fold upregulation takes place in astrocyte gene *PTX3*, a key regulator of neurogenesis and angiogenesis after cerebral ischemia (. Rodriguez-Grande et al., J Neuroinflammation 12:15, 2015); and (c) *Tmem119* and *P2y12*, two markers of homeostatic microglia, were found to be enhanced by ten- and fivefold, respectively (Walker et al. Int J Mol Sci 21:678, 2020). Overall, we uncovered that protection after middle cerebral artery occlusion (MCAo) by the lipid mediators elicits expression of microglia and astrocyte-specific genes (*Tmem119, Fcrls, Osmr, Msr1, Cd68, Cd163, Amigo2, Thbs1,* and *Tm4sf1*) likely participating in enhancing homeostatic microglia, modulating neuroinflammation, promoting DAMP clearance, activating NPC differentiation and maturation, synapse integrity and contributing to cell survival.

**Graphical Abstract:**

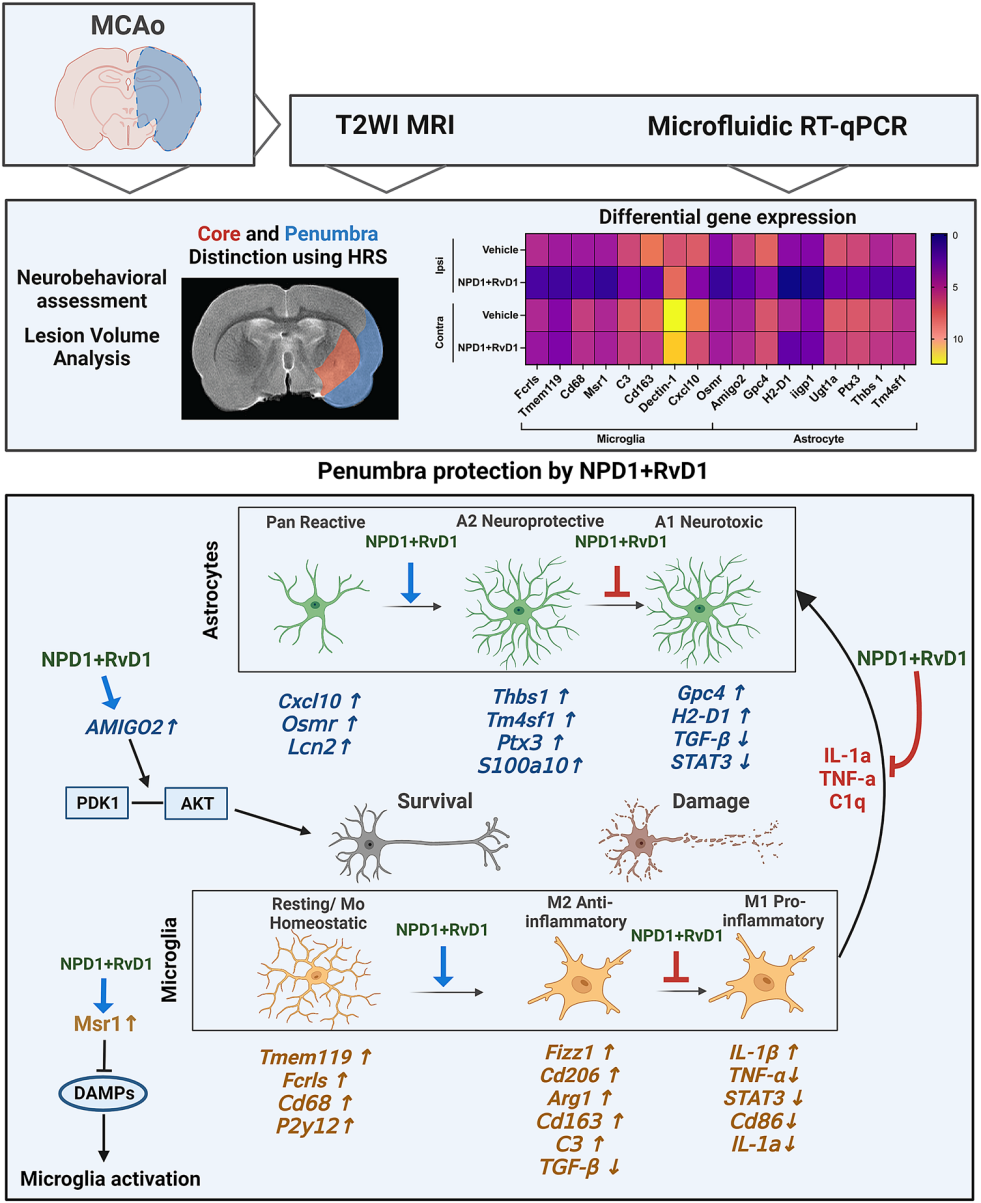

**Supplementary Information:**

The online version contains supplementary material available at 10.1007/s10571-023-01363-3.

## Introduction

Stroke is a leading cause of death and disability worldwide (Guzik and Bushnell 2017). The treatment of ischemic stroke is to administer tissue plasminogen activator (t-PA) within 4.5 h of stroke onset, and only 5–8% of patients qualify for this therapy (Liaw and Liebeskind 2020). A recently completed EXTEND trial showed that this could be extended up to 9 h after stroke onset guided by CT or MRI perfusion images, thus allowing more patients eligible to receive tPA beyond the 4.5-h time window (Ma et al. 2019a; Campbell et al. 2019). Despite active stroke research, all treatments have failed to show favorable clinical outcomes. Multi-targeted drug therapy holds more promise than single-class drug regimens (Lapchak 2011) since some drug combinations exhibit pharmacological synergism, translating into lower doses, fewer adverse side effects, and extending the therapeutic window (Saleh et al. 2014). Omega-3 polyunsaturated fatty acids (PUFAs) have been proposed as beneficial for cerebrovascular diseases, including stroke, carotid stenosis, vertebral and intracranial stenosis, aneurysms, and vascular malformations (Marcheselli et al. 2003; Miao et al. 2021). They exert multiple biological functions, including limiting excessive inflammatory responses, regulating metabolism and immune cell functions, decreasing the production of pro-inflammatory factors, increasing anti-inflammatory mediators, and promoting tissue repair and homeostasis (Miao et al. 2021).

Inflammation is a prime target to modulate because it can be both detrimental and beneficial after stroke (Marcheselli et al. 2003; Kelly et al. 2021). Moreover, the resolution of inflammation is not passive as it engages a biosynthetically active process, regulated by specific mediators and receptor-signaling pathways and driven by specialized pro-resolving mediators (SPMs) (Serhan and Petasis 2011). Pro-resolving lipid mediators derived from eicosapentaenoic acid (EPA) and DHA were identified (Marcheselli et al. 2003; Serhan et al. 2004; Mukherjee et al. 2004; Spite and Serhan 2010), including Neuroprotectin D1 (NPD1) and D-series resolvins (RvD1), which display potent anti-inflammatory and immunoregulatory properties (Asatryan and Bazan 2017). They antagonize pro-inflammatory signaling on macrophage/neutrophil infiltration, enhance macrophage phagocytosis towards apoptotic cells, and accelerate tissue repair (Cheng and Rong 2019). The neuroprotective action of NPD1 in focal cerebral ischemia (Marcheselli et al. 2003; Belayev et al. 2018) promotes cell survival, inhibits brain ischemia–reperfusion (I/R)-mediated leukocyte infiltration and pro-inflammatory gene expression, promotes neurogenesis, attenuates edema formation, and reduces stroke volume continuously delivered into the third ventricle during 48 h after middle cerebral artery occlusion (MCAo) in mice (Rodriguez de Turco et al. 2002; Marcheselli et al. 2003). In addition, LC–MS/MS-based mediator lipidomic analysis of the ipsilateral cortical region demonstrated the synthesis of NPD1 following DHA treatment in the ischemic penumbra at 5 h after MCAo (Belayev et al. 2018). NPD1 also upregulates the anti‐apoptotic proteins Bcl‐2 and Bcl-xL and decreases pro‐apoptotic Bax and Bad expression (Bazan 2005). Additionally, it inhibits oxidative stress‐induced caspase‐3 activation and IL‐Iβ‐stimulated expression of COX‐2 and protects cells from oxidative stress‐induced apoptosis (Bazan 2009). In addition, NPD1 administered into the right lateral ventricle improves behavior, reduces lesion volumes, protects the ischemic penumbra, and increases NeuN, GFAP, SMI-71-positive cells and vessels, axonal regeneration in the penumbra, and BBB after MCAo in rats (Belayev et al. 2018).

Resolvins are derived from DHA or EPA, and after their release from the cell membrane, the enzymes LOXs and cytochrome-P450 convert DHA to the D-series resolvins (RvD1–RvD6) (Videla et al. 2019; Ostermann et al. 2019). DHA-derived D-series resolvins also reduce inflammation in kidney injury and cardiovascular and autoimmune disorders (Serhan 2017). RvD1 and RvD2 have been thus far studied in relation to stroke (Bisicchia al. 2018). Both have similar structures but distinct receptors, i.e., RvD1 exerts its actions through FPR2 or GPR32, whereas GPR18 mediates the activity of RvD2 (Krishnamoorthy et al. 2010). RvD1 IP administered improved functional recovery and protected hemicerebellectomy-lesioned rats against remote neuronal cell death and neuroinflammation (Bisicchia et al. 2018). Together, NPD1 and RvD1 thus possess potent anti-inflammatory effects that could mitigate the adverse outcomes after stroke.

D-series resolvins have multiple beneficial actions (Spite and Serhan 2010) by reducing excessive polymorphonuclear leukocyte (PMN) infiltration, decreasing PMN activation, and promoting phagocytosis and clearance of apoptotic cells (Krishnamoorthy et al. 2010) and in acute and chronic inflammatory diseases, such as peritonitis and sepsis (Spite and Serhan 2010). In addition, resolvins are beneficial in inflammation resolution following fluid-percussion injury, assessing post-traumatic sleep, and cognitive and sensorimotor outcomes (Harrison et al. 2015). RvD1 is an endogenous anti-inflammatory lipid mediator involved in neurodegenerative diseases as well as in atherosclerosis (Miao et al. 2021) that improved plaque stability in fat-fed Ldlr ^−^/^−^ mice via increased fibrous caps and suppressed necrosis and inflammation (Fredman et al. 2016).

Because microglia and astrocytes are activated at stroke onset and participate in post-stroke inflammatory signaling producing cytokine and promoting leukocyte infiltration (Jayaraj et al. 2019), we assessed the expression of astrocyte and microglial genes to understand potential mechanisms of NPD1 and RvD1-mediated protection. Microglia activated after ischemic stroke undergoes morphologic and phenotypic changes reflecting the severity of the ischemic injury (Emmrich et al. 2015). In ischemic stroke, however, activated microglia serve dual functions secreting both pro- and anti-inflammatory factors; thus, inhibition of microglial activation and over-activation would contribute to stroke-associated brain damage (Jayaraj et al. 2019). Microglia in the peri-infarct zone are activated after MCAo onset, and an increase in markers CD11b, CD45, and Iba1 is observed (Clausen et al. 2008; Xu et al. 2020). Phenotypic changes in microglia can be classified into a pro- or anti-inflammatory type, M1 and M2, respectively. While this is an oversimplification of microglia classification, as demonstrated by transcriptomics, microglial polarization into sub-types is temporal in stroke pathophysiology. For example, the M2 microglia can be further subdivided into M2a, M2b, and M2c phenotypes based on different functions. Damage-associated molecular pattern molecules (DAMPs), including high-mobility group box 1 (HMGB1), are the main factor that activates microglia. Several other factors also influence ischemia, such as toll-like receptors (TLRs), receptors for chemokines and cytokines, P2y12 and P2X7 purinergic receptors, and trigger receptors expressed on myeloid cells 2 (TREM2). These factors are involved in many signal transduction pathways, for example, MAPK, NF-kb, and PPAR. M1 and M2 microglia can be distinguished by looking at the cells’ biological function and their secreted chemokines and cytokines. Following the acute phase of stroke, the inflammatory response declines gradually, and tissue repair begins, where microglia function to modulate neurogenesis to replace the injured cells by influencing cell migration and synaptic activity (Ji et al. 2013; Hiu et al. 2016). In the acute stages of stroke (within 1 day), microglia proliferation and activation can cause a robust inflammatory response detrimental to the central nervous system; however, in the chronic stages (several days after stroke onset), the production of protective cytokines by microglia contributes to repair and cell survival (Xu et al. 2020).

Astrocyte activation refers to the phenotypic changes, hyperplasia, and swelling when astrocytes transform from resting to reactive state, causing an increase in reactive astrocyte proteins: *GFAP*, *S100beta*, and vimentin (Shen et al. 2021). Astrocytes activation may also be related to the release of some inflammatory factors by microglia, such as transforming growth factor-alpha (*TGF-a*), interleukin-6 (*IL-6*), Clq, interleukin-1a (*IL-1a*), and tumor necrosis factor (*Tnf-a*) (Liddelow et al. 2017). Key signaling pathways and transcription factors are closely related to astrocyte activation, including JAK/STAT3 and TGF-B/Smad signaling. However, activated astrocytes can also play a positive role in ischemic stroke, protecting against oxidative stress, releasing neurotrophic factors, reducing cerebral edema, and protecting neurons (Shen et al. 2021).

The present study assessed whether NPD1, RvD1, and their combination exert neuroprotection when systemically administered in different doses up to 6 h after focal cerebral ischemia in rats. In addition, we investigated whether the administration of NPD1 + RvD1 affects the expression of microglia and astrocyte-specific genes. We used magnetic resonance imaging in conjunction with RT-qPCR and behavioral testing to enhance our understanding of the neuroprotection elicited by the lipid mediators.

## Materials and Methods

### Animals and Surgical Preparation

All experimental protocols were approved by the Institutional Animal Care and Use Committee (IACUC) of the Louisiana State University Health Sciences Center, New Orleans (IACUC protocol number 3778). Before the surgical procedure, male Sprague–Dawley (SD) rats housed 2 per cage prior to surgery (270–320 g; Charles River Lab, Wilmington, MA, USA) were fasted overnight and allowed free access to water. Inhalation of 3% isoflurane in 70% NO and 30% O_2_ mixed gases was used to induce anesthesia, maintained with 1% isoflurane in the same gas mixture during the procedure. Animals were immobilized with pancuronium bromide (0.6 mg/kg, IV), orotracheally intubated, and mechanically ventilated. Catheters were implanted into the right femoral artery and vein. Before, during, and after the procedure, serial analyses of arterial blood gases, plasma glucose, blood pressure, and heart rate were conducted. PCO2 was maintained at 36–40 mmHg and PO2 at 105–120 mmHg, adjusting via a ventilator. Rectal temperature was closely monitored and held with a heating lamp and plate at 36–37.5 °C. The cranial temperature was measured by a needle microprobe under the temporalis muscle and regulated at 36.2–37 °C. Rats were housed 1 per cage after surgery.

### Transient Middle Cerebral Artery Occlusion (MCAo)

Conducted as previously published (Belayev et al. 1996). Briefly, a midline incision in the neck exposed the right common carotid artery (CCA), dissected to free it from surrounding nerves and fascia. A monofilament nylon suture coated with a poly-L lysine was inserted via the proximal external carotid artery into the internal carotid artery and advanced at 21–22 mm distal from the bifurcation of the CCA to block the origin of MCAo. After 60 min of MCAo, rats were tested on a neurologic battery (0 = normal and 12 = maximal deficit) to confirm high-grade neurologic deficit (Belayev et al. 1996). Only rats with a high-grade deficit (10 or greater) were used. After 2 h of MCAo, rats were re-anesthetized with the same anesthetic combination, intraluminal sutures were removed, and the animals were allowed to recover for 7 days with free access to food and water.

### Treatments and Experimental Protocols

NPD1 and RvD1 (Cayman Chemical Co, Ann Arbor, Michigan, USA) were dissolved in 0.9% saline and administered (IV) into the femoral vein at 3 h after stroke onset at a constant rate over 3 min using an infusion pump. NPD1 was administered first at 3 h and then RvD1 15 min later for combinatory treatment. Vehicle (0.9% saline) was administered at 3 h. All experiments were done by researchers blinded to the treatment groups.

#### Dose-Response

Animals were randomly assigned to eight groups: NPD1 (111, 222, or 333 µg/kg), RvD1 (111, 222, or 333 µg/kg), NPD1+RvD1, and saline. Behavior testing was conducted, followed by an ex vivo MRI on day 7. *n* = 4–10 in each group.

#### Therapeutic Time Window

Animals were randomly assigned to seven groups: Vehicle, NPD1 (222 µg/kg), or RvD1 (222 µg/kg) administered at 3 h, NPD1+RvD1 at 3, 4, 5, and 6 h after onset of MCAo. Behavior testing was conducted, followed by an ex vivo MRI on day 7. *n* = 5–10 in each group.

#### RT-qPCR Gene Expression

A separate animal cohort was assigned to two groups: Vehicle or NPD1+RvD1 (222 µg/kg) administered at 3 h after the onset of MCAo. The behavioral evaluation was conducted, followed by brain sampling of the ischemic core and penumbra at 24 h. *n* = 5–9 in each group.

### Assessment of Functional Outcomes

The experiments were performed between 8:00 am and 4:00 pm by researchers blinded to the treatment groups. Rats underwent neurobehavioral testing before MCAo and on days 1, 2, 3, and 7 after MCAo. All tests were performed by an investigator blinded to the treatment groups. A neurological battery consisted of two components: (1) a postural reflex test, designed to examine forelimb and upper-body posture in response to tail-suspension and lateral displacement, regarded as being sensitive to both cortical and striatal lesions; and (2) an elicited forelimb placing test, which examines sensorimotor integration by assessing placing reactions to visual, tactile, and proprioceptive stimuli. The total neurologic score is graded on a scale of 0 (no deficit) to 12 (maximal deficit), as we previously described (Belayev et al. 1996).

### Magnetic Resonance Imaging (MRI) Acquisition and Analysis of Total Lesion, Core, and Penumbra Volumes

High-resolution ex vivo MRI was conducted on 4% paraformaldehyde-fixed brains on day 7 using an 11.7 T Bruker Advance (Bruker Biospin, Billerica, Massachusetts, USA). T2-weighted imaging (T2WI) and T2 relaxation maps were computed as we previously described (Ghosh et al. 2012). Hierarchical Region Splitting (HRS) was used to automatically identify core and penumbra volumes (total lesion = core + penumbra) from T2 relaxation maps (Ghosh et al. 2012). Our core and penumbral tissue determination using HRS (implemented in Matlab) have been previously validated using perfusion-weighted imaging (PWI)/diffusion-weighted imaging (DWI) subtractions at each brain level (Ghosh et al. 2012). The penumbra from HRS was defined using T2 values (ms) between normal-appearing brain tissue and the ischemic core. Data were summarized per group.

### High-Density qPCR (HT-qPCR)

#### Primer Selection and Development

Seventy-six transcripts of differentially expressed genes following ischemic stroke were selected for analysis (Table S1). Candidate genes were selected from a literature search to determine stroke-associated astrocyte and microglia subtype-specific genes that have been shown to be differentially expressed following stroke or play a role in stroke pathophysiology (Jurga et al. 2020; Androvic et al. 2020; Williamson et al. 2021; Dong et al. 2021; Li et al. 2022).

#### Sample Collection and RNA Isolation

Brain samples of the anterior and posterior ipsilesional cortex and subcortex regions were harvested 24 h after reperfusion, flash-frozen in liquid nitrogen, and stored at − 80 °C until use. Tissue samples from each region were homogenized using TRIzol® reagent (Cat. #15596026 l, Invitrogen, Thermo Fisher Scientific, Inc., Waltham, MA, USA) according to the manufacturer’s protocol. Total RNA was prepared using RNeasy columns (Cat. #74004, Qiagen, Valencia, CA). RNA quantity and purity were assessed using the NanoDrop One spectrophotometer (Thermo Scientific). Optimal purity of RNA was ensured by determination of the 260/280 adsorption ratio (values > 2.00).

#### Reverse Transcription

One microgram of total RNA was reverse-transcribed per sample into first-strand complementary DNA (cDNA). cDNA samples were stored at − 20 °C for a maximum of 4 weeks.

#### Specific Target Amplification (STA)

To ensure adequate amounts of templates of the target genes for the high-throughput qPCR, a specific target gene amplification (STA) was performed. For STA, all sequence-specific primer pairs of the target genes were pooled and diluted with DNA suspension buffer to a final concentration of 500 nM (pooled primer mixture). Stock solutions of the pooled primer mixture were stored at − 20 °C. A total of 5 µL STA mix was prepared to contain 2.5 µL 2×PreAmp Master Mix, 0.5 µL of the 500 nM pooled primer mixture, 0.75 µL PCR-certified water, and 1.25 µL cDNA per reaction. A PCR-certified water control (NTC-STA) and a non-reverse-transcribed RNA (NoRT) control were also included. STA was performed in a thermal cycler (Mastercycler ®nexus, Eppendorf) using the following temperature program: 10 min at 95 °C as an initial denaturation step followed by 12 cycles of 15 s at 95 °C for denaturation and 4 min at 60 °C for annealing and elongation and a final holding temperature of 4 °C. To prevent the carry-over of unincorporated primers after the STA reaction, samples were treated with exonuclease I (Escherichia coli; Cat. #M0293S, New England Biolabs). To the STA samples, 2 µL of the exonuclease reaction mixture was added, and digestion with Exo I at 4 units/µL was performed in a thermal cycler with the following temperature program: 40 min at 37 °C for digestion of the unincorporated primers and dNTPs, 15 min at 80 °C to inactivate Exo I and a final holding temperature at 4 °C. STA and Exo I-treated samples were diluted tenfold with 43 µL TE buffer.

#### Preparation of Samples and Primers

Forward and reverse primers (100 µM) were diluted to 5 µM by adding 2.5 µL of each primer pair to 25 µL of 2×Assay Loading Reagent and 22.5 µL of DNA suspension buffer. The primer reaction mix was stored at − 20 °C. For the sample mix, 2.25 µL of STA and Exo I-treated samples were mixed with 2.5 µL of 2×SsoFast™ EvaGreen® Supermix with Low ROX and 0.25 µL of 20×DNA Binding Dye Sample Loading Reagent (Cat. #1725211; Bio-Rad).

#### High-throughput RT-qPCR

HT RT-qPCR was run on the BioMark HD System, using 96×96 Fluidigm Dynamic Arrays (Cat. #BMK-M-96.96; Fluidigm, South San Francisco, CA). Preparation and loading of Fluidigm 96.96 Dynamic Array IFC (integrated fluidic circuit) were performed according to the manufacturer’s instructions. Preparation of the 96.96 Dynamic Array IFC included the injection of 150 µL of a control line fluid into each chip accumulator with a syringe. The chip was then placed into the Juno and run with the prime 96.96 GE. After priming, the chip was loaded with samples, and the primer reaction mixes within 1 h to reduce the pressure loss within the chip. Thus, 5 µL of each primer reaction mix and each sample were loaded into respective inlets. Samples and primer reaction mixes were loaded into the chip into the Juno and running the Load mix 96.96 GE script. The chip was transferred into the BioMark™ HD System, and qPCR and melting curve analysis were performed by running the following temperature program: 2400 s at 70 °C and 30 s at 60 °C, followed by a hot start for 60 s at 95 °C, 30 PCR cycles of 5 s at 96 °C for denaturation and 20 s at 60 °C for annealing and elongation. The melting curve analysis consisted of 3 s at 60 °C followed by heating up to 95 °C with a ramp rate of 1 °C/3 s.

#### RT-qPCR Data Analysis

Raw data were pre-processed with the Real-Time PCR analysis software v4.1.3 (Fluidigm); unspecific values were deleted based on melting-curve analysis. The relative mRNA expression levels of predicted key genes were calculated using the 2 − ΔΔCT method, following normalization to the geometric mean of housekeeping genes: ACTB, GAPDH, GUSB, PGK1, PP1A, RP13A, and TFRC.

#### Network Establishment and Analysis

A dataset for each brain region was constructed and imported into the IPA system for data visualization and detailed analysis. The network analysis algorithm was based on Fisher’s exact test with the enrichment score of P-values. The genes with known gene IDs (Ensembl) and their corresponding expression values were uploaded into the software. Each gene symbol was mapped to its corresponding gene object in the Ingenuity Pathways Knowledge Base. Networks of these genes were algorithmically generated based on their connectivity and assigned a score. The score is a numerical value used to rank networks according to how relevant they are to the genes in the input dataset, but it may not be an indication of the quality or significance of the network. The score considers the number of focus genes in the network and the size of the network to approximate how relevant this network is to the original list of genes. IPA utilizes four distinct causal algorithms: (1) Upstream Regulator Analysis (URA) identifies potential regulators; (2) Mechanistic Networks (MN) enhance URA by linking regulators likely involved in the same signaling or causal mechanism within hypothesis networks; (3) Causal Network Analysis (CNA) expands upon URA by connecting upstream regulators to dataset molecules via paths with more than one link (e.g., through intermediate regulators), generating a comprehensive view of potential root causes for observed expression changes; and (4) Downstream Effects Analysis (DEA) applies URA principles to deduce and evaluate the influence on biological functions and diseases that are downstream of genes with altered expression in a dataset. (Krämer et al. 2014). The networks identified are then presented as a graph indicating the molecular relationships between genes/gene products. The “path designer” module was used to polish the network images and graphs.

### Statistical Analysis

Sample size for each group in the experiments was determined using a power analysis. A priori power analysis was conducted using G*Power version 3.1.9.7 (Faul et al., 2007) for sample size estimation based on Neurologic data from a previous study (Belayev et al. 2018). With a significance criterion of *α* = 0.05 and power = 0.80, the minimum sample size needed with this effect size is *N* = 3 for Wilcoxon-Mann Whitney test of two groups. Thus, the obtained sample size of *N* = 4–10 for both dose response and therapeutic window studies is more than adequate. For gene expression studies, the same parameters for alpha and power were used, and group means and SD were determined from a pilot study using the same methodology described above. The minimum sample size needed with this effect size is *N* = 4 for Wilcoxon-Mann Whitney test of two groups. Data from different experiments were presented as mean ± SEM for non-parametric data and mean ± SD for parametric data as indicated. Normality was assessed in GraphPad Prism using the Kolmogorov–Smirnov test for normality, and statistical testing was performed using RStudio 2021.09.0. Mann–Whitney non-parametric tests were used for two-group comparisons of all neurologic data. Dunnet’s multiple comparison tests were used for all bregma distribution data if normally distributed. Wilcoxon-Mann–Whitney tests were used to compare groups for gene expression ∆Ct values. Data were considered significant when *p ≤ 0.05, **p ≤ 0.01, ***p ≤ 0.001, ****p ≤ 0.0001. All statistics were analyzed and graphed with the GraphPad Prism9 package.

## Results

Physiological variables were defined in all experimental groups, and no adverse behavioral side effects were observed after NPD1, RvD1, or NPD1 + RvD1 administration. Four animals died during the dose–response experiment: two rats in the saline group on days 2 and 3, one from each group NPD1 (333 µg/kg) and RvD1 (222 µg/kg) on days 2 and 3, respectively. Two rats died during the therapeutic window study: saline and NPD1 + RvD1 groups (both at 24 h). Autopsies revealed large ipsilateral hemispheric infarctions and extensive brain edema in all rats.

### NPD1 + RvD1 Dose–response and Therapeutic Window Demonstrate Neuroprotection by Improving Behavior and Decreasing T2WI Stroke Lesion

#### Dose–Response

**Fig. 1:**
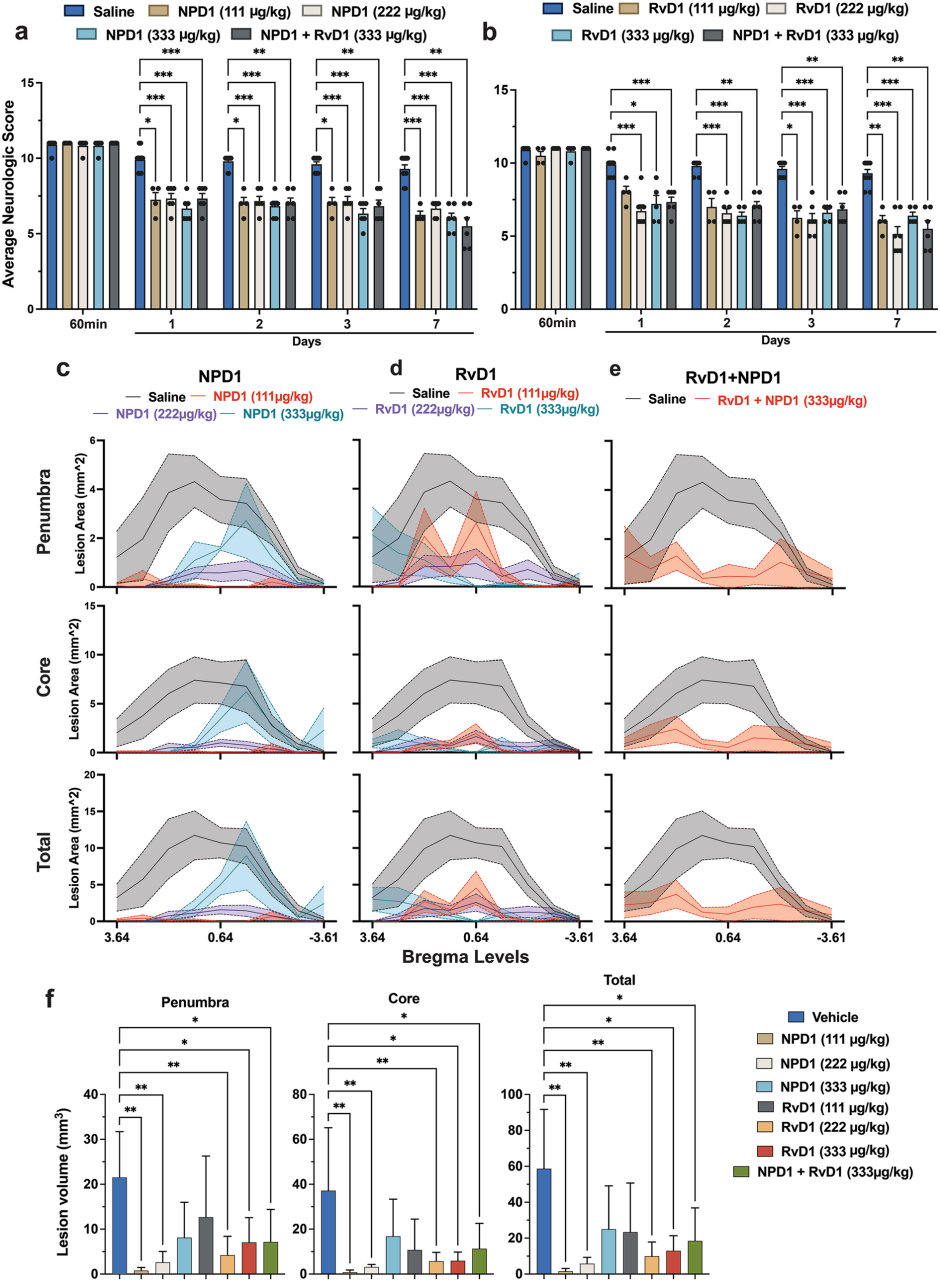
Dose–response shows that NPD1, RvD1, and their combination improved functional recovery after MCAo. (**a-b**) Total neurologic score (normal = 0, maximal deficit = 12) during 60 min of MCAo and 1, 2, 3, and 7 days after MCAo. All treatments improved total neurological scores compared to the vehicle group. Data are mean ± SEM, *n* = 8–6 per group. *p ≤ 0.05, **p ≤ 0.01, ***p ≤ 0.001, ****p ≤ 0.0001 versus saline group (repeated-measures ANOVA followed by Bonferroni tests). The distribution of penumbra, core, total lesion areas (**c-e**), and volumes (**f**) were computed from T2WI on day 7. NPD1, RvD1, and their combination protected the ischemic core, penumbra, total lesion, and volume after stroke. Data are mean ± SEM, n = 8–6 per group. Statistics for **c–e** are reported in Table S2

The study to evaluate the most efficacious dosage of NPD1 and RvD1 showed improvements in neurological deficit compared to the vehicle for NPD1 dosages of (111, 222, and 333 µg/kg) and RvD1 (222 and 333 µg/kg) as well as improvements when NPD1 + RvD1 were administered in combination (Fig. 1a–b**;** Fig. S1a–b**;** Table S2). The bregma level lesion distribution from T2WI MRI imaging shows reduction for all groups compared to saline (Fig. 1c–e**; **Table S3), and lesion volume was reduced significantly by NPD1 (111, 222, and 333 µg/kg), RvD1 (222, 333 µg/kg), and their combination in the ischemic penumbra. Lesion volume reduction in the ischemic core was only observed by NPD1 and RvD1 (222 µg/kg) (Fig. 1f).

#### Therapeutic Window

**Fig. 2:**
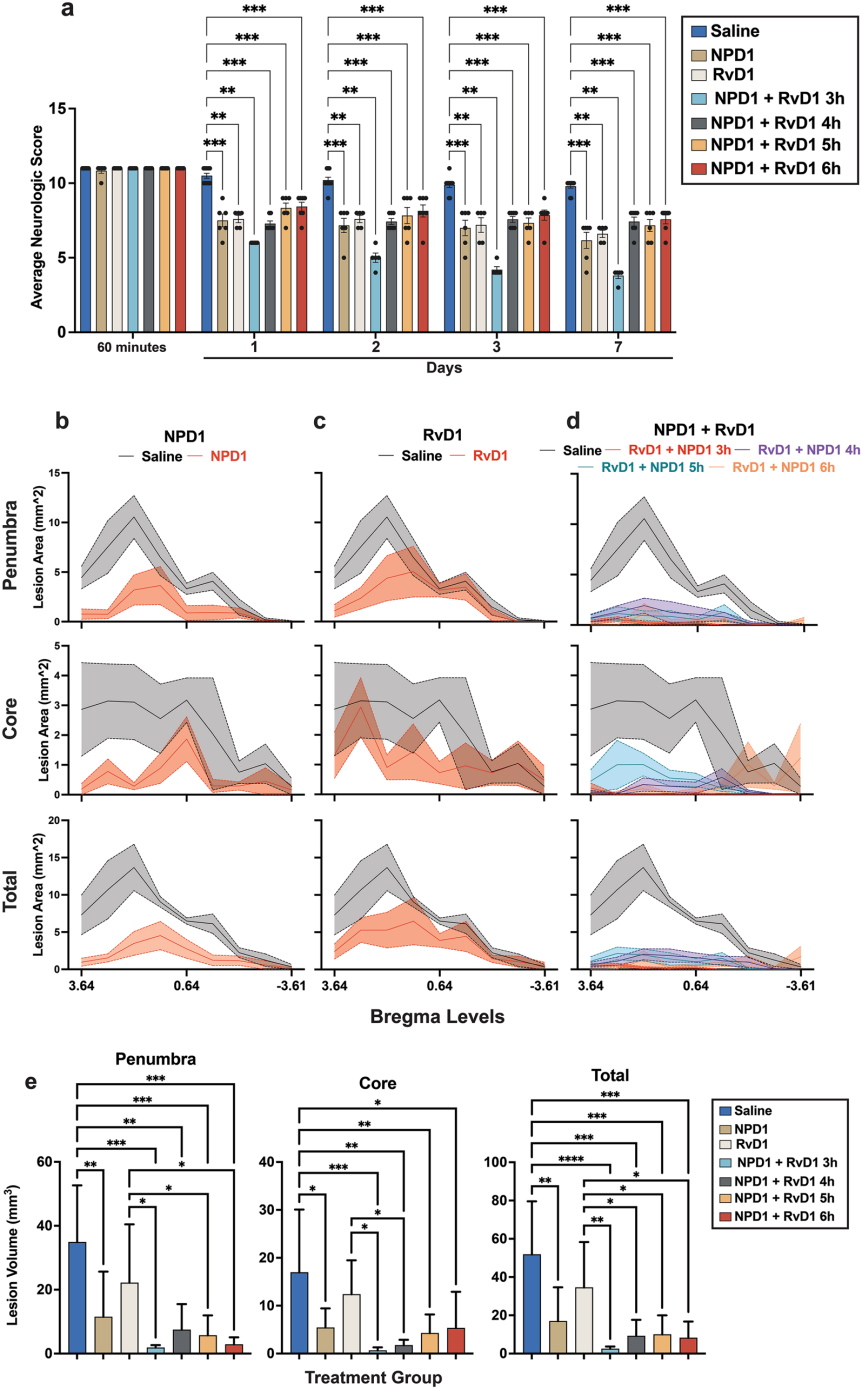
Therapeutic window uncovers that NPD1, RvD1, and their combination improved neurologic deficits when administered up to 6 h after MCAo. (**a**) Total neurologic score (normal = 0, maximal deficit = 12) during 60 min of MCAo and 1, 2, 3, and 7 days after MCAo. Graphs show improvement in total neurological score in all treated groups vs. saline-treated rats. Behavioral data are means ± SEM; *n* = 5–6 per group. (**b–d**) The distribution of penumbra, core, total lesion areas, and (**e**) volumes were computed from T2WI on day 7. Treatment with NPD1 + RvD1 decreased core, penumbra, and total lesion areas and volumes when administered up to 6 h after stroke. Statistics for **b**–**d** are reported in Table S3. Data are mean ± SEM; *n* = 5–6 per group, *p ≤ 0.05, **p ≤ 0.01, ***p ≤ 0.001, ****p ≤ 0.0001 versus saline group (Mann–Whitney test)

Investigation of the therapeutic window showed that treatment with NPD1 and RvD1 alone, when administered only at 3 h, improved total neurological score on days 1, 2, 3, and 7 compared to vehicle (Fig. 2a). The neuroprotective effect was enhanced by combinatorial NPD1 + RvD1 treatment, which improved behavior by 61% when administered at 3 h, 26% at 4 h, 25% at 5 h, and 31% at 6 h (Fig. 2a). The combinatory treatment administered at 3 h was significantly better than NPD1 or RvD1 alone at every observation point throughout the 7-day survival period (Fig. 2a; Table S4). Penumbra, core, and total lesion areas and volumes computed from T2WI on day 7 presented in Fig. 2b–e. Ischemic core, penumbra, and total lesion areas were reduced on multiple levels by all treatments compared to saline when administered up to 6 h after the onset of MCAo (Fig. 2b–d; Table S5, S8). Compared to the saline group, the combinatory treatment administered at 3, 4, 5, and 6 h reduced lesion volumes in the ischemic penumbra and total lesion. Ischemic core volumes were also reduced by combinatory treatment, administered at 3 and 4 h. The combinatory treatment administered at three h was significantly better than RvD1 alone in reducing penumbra, core, and total volumes on day 7 (Fig. 2e, Table S9). The combinatory treatment administered at 4 h was significantly better than NPD1 alone in reducing core volumes (Fig. 2e). Although lesion volumes were reduced in NPD1 or RvD1 treated groups alone, there were no statistical differences compared to saline-treated animals (Fig. 2e).

### NPD1 + RvD1 Modulates Gene Expression of Astrocytes and Microglia While Protecting the Penumbra After Ischemic Stroke

**Fig. 3:**
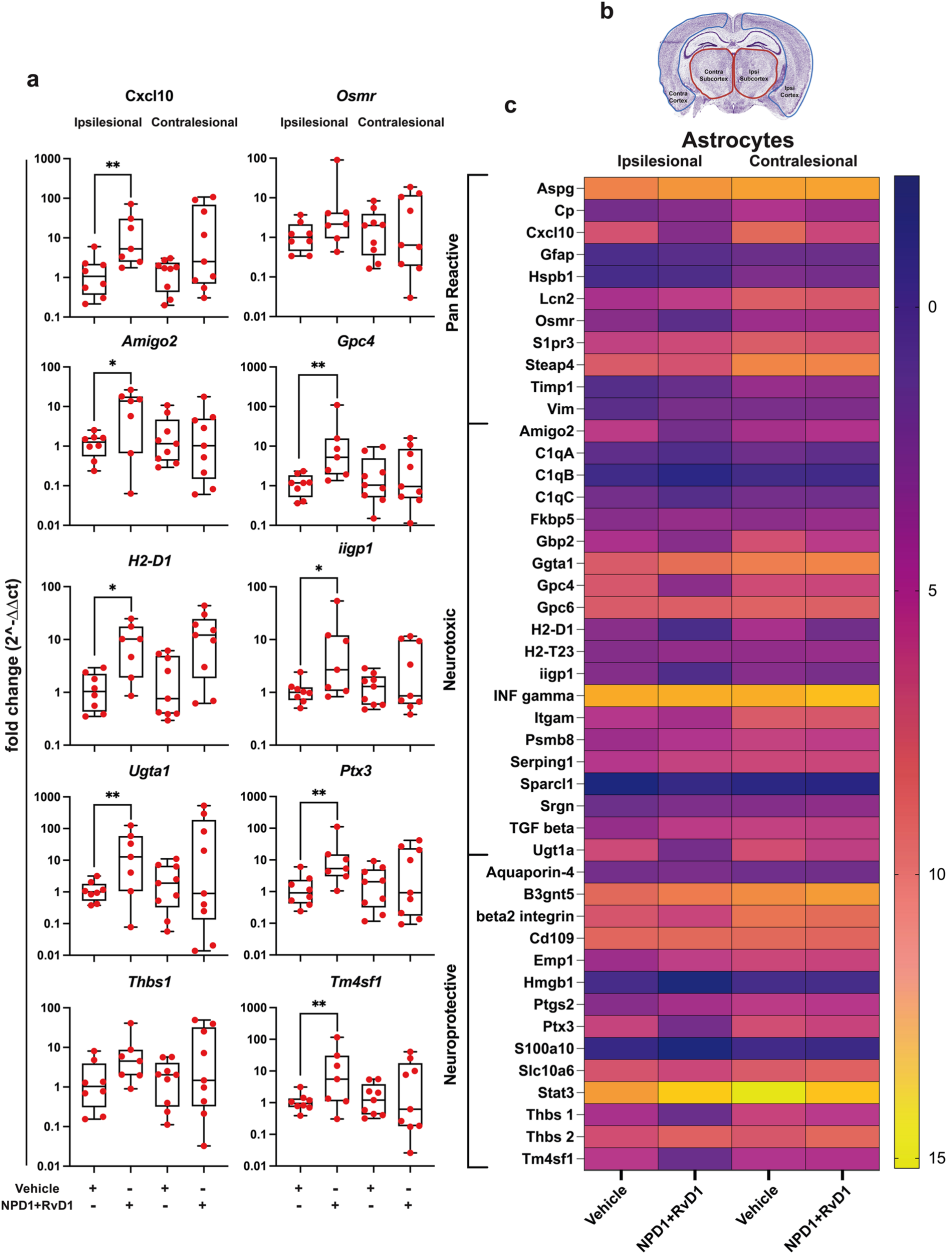
NPD1 + RvD1 mediated ipsilesional penumbra protection correlates with selective astrocyte gene expression. (**a**) Boxplots of the most differentially expressed astrocyte genes in NPD1 + RvD1 treated rats at 24 h after MCAo. Data are presented as fold change (2^-∆∆Ct) compared to the control. (**b**) Heatmap of mean ∆Ct values of investigated genes in the vehicle and NPD1 + RvD1 treated rats in ipsilesional and contralesional for comparison. *n* = 6–9 per group, *p ≤ 0.05, **p ≤ 0.01, ***p ≤ 0.001, ****p ≤ 0.0001 versus saline in the respective brain region (Multiple t-tests or Mann–Whitney)

Exploring the mechanism of penumbra protection provided by NPD1 + RvD1 was achieved by analyzing 76 target genes related to microglia and astrocyte in ipsilesional and contralesional cortex and subcortex brain regions of rats treated with the combination of lipid mediators or saline. A heatmap of normalized ∆Ct values in the ipsilesional penumbra is shown for all 45 astrocyte targets, with significant gene fold change boxplots (Fig. 3a–b; Table S6, S7). Figure 3a depicts the upregulation of homeostatic and neuroprotective astrocyte markers by NPD1 + RvD1 in the ipsilesional penumbra. Pan-reactive astrocyte genes *Cxcl10* and *Osmr* increased 19- and 15-fold change, respectively*.* Neurotoxic astrocyte genes *Amigo2*, *Gpc4, H2-D1, Iigp,* and *Ugt1a* increased by a fold change of 12, 20, 10, 12, and 33, respectively. Neuroprotective astrocyte genes *Ptx3, Thbs1*, and *Tm4sf1* were also upregulated 21-, 9-, and 24-fold.

**Fig. 4:**
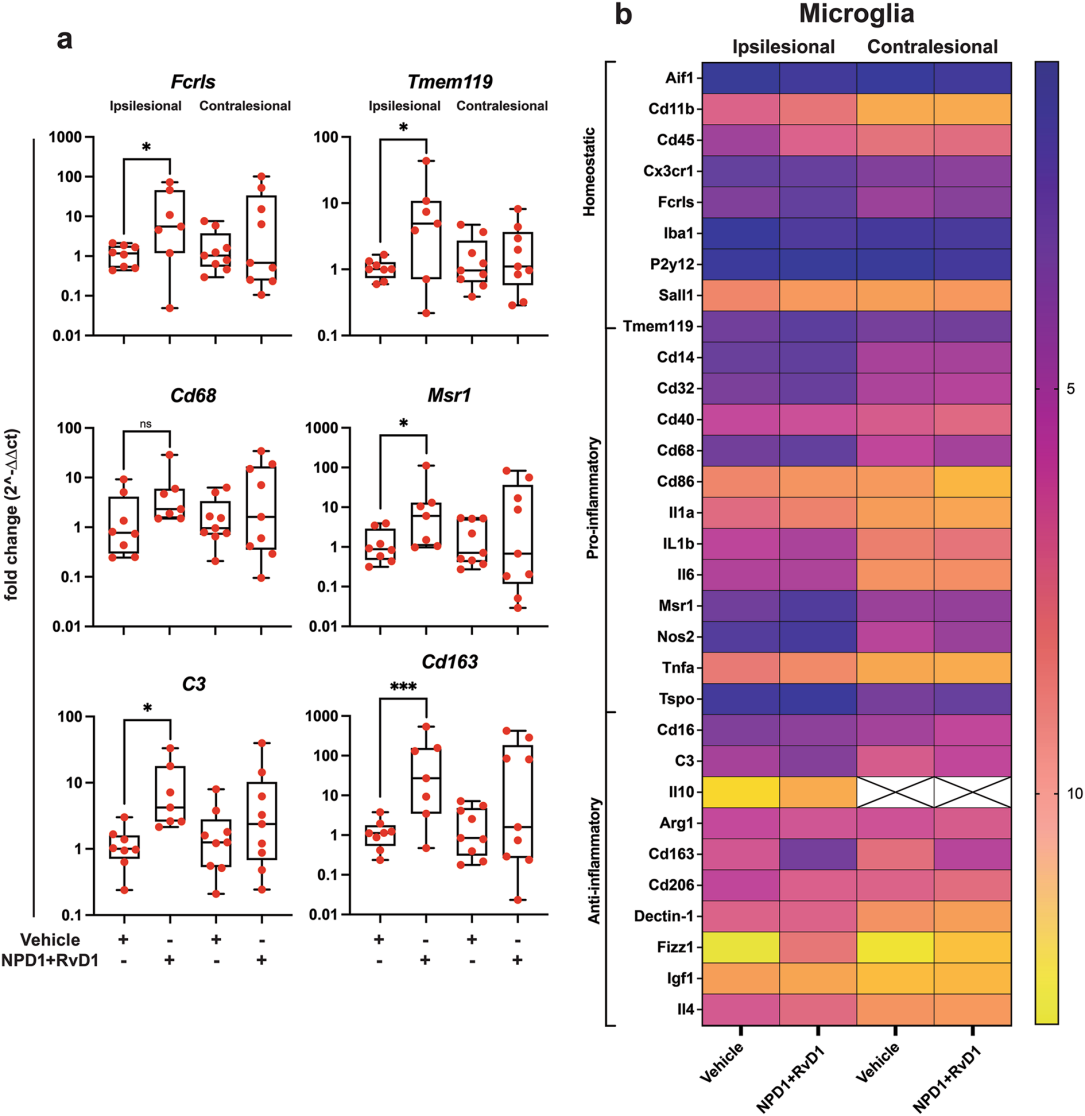
NPD1 + RvD1 mediated ipsilesional penumbra protection correlates with selective microglial gene expression. (**a**) Boxplots of the most differentially expressed microglia genes in NPD1 + RvD1 treated rats at 24 h after MCAo. Data are presented as fold change (2^-∆∆Ct) compared to the control. (**b**) Heatmap of mean ∆Ct values of investigated genes in the vehicle and NPD1 + RvD1 treated rats in ipsilesional and contralesional for comparison. *n* = 6–9 per group, *p ≤ 0.05, **p ≤ 0.01, ***p ≤ 0.001, ****p ≤ 0.0001 versus saline in the respective brain region (Multiple t-tests or Mann–Whitney)

Microglia targets were also investigated to determine the effect of NPD1 + RvD1 on microglia gene expression. A heatmap of normalized ∆Ct values from the ipsilesional penumbra is shown in Fig. 4b. Significant gene fold-changes are plotted in Fig. 4a**.** Homeostatic microglia genes *Fcrls* and *Tmem119* increased 20- and tenfold, respectively. Pro-inflammatory microglia genes *Cd68* and *Msr1* were increased 7- and 21-fold, and anti-inflammatory genes *C3* and *Cd163* 10 and 124-fold. The changes indicate that the bioactive lipids mediate the expression of homeostatic and anti-inflammatory microglia markers and decrease the expression of pro-inflammatory markers. This could suggest that NPD1 + RvD1 modulates the polarization of M1 phenotypes in the ipsilesional penumbra (Fig. 4a).

### Functional Analysis of NPD1 + RvD1 Mediated Gene Expression Indicates Inhibition of Cell Death Signaling and Modulation of Stroke-associated Signaling Pathways

**Fig. 5:**
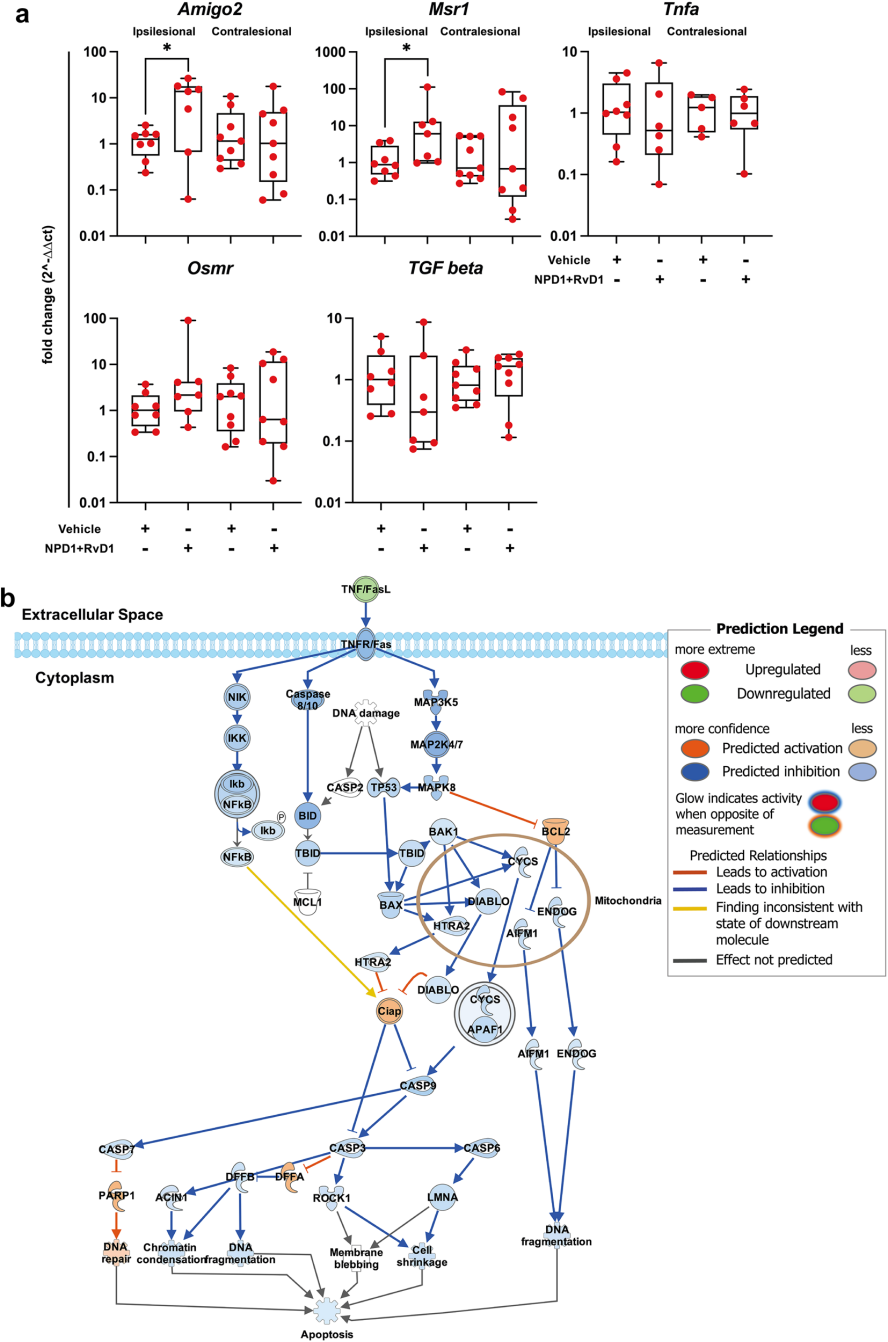
NPD1 + RvD1 inhibits cell death signaling, providing penumbral protection after MCAo. (**a**) Boxplots with the expression levels of cell death related genes. Data are presented as fold change (2^-∆∆Ct) compared to the control. (**b**) Apoptosis pathway generated by IPA with an overlay of predicted relationships from NPD1 + RvD1 treated samples of the ipsilesional penumbra. Treatment with NPD1 + RvD1 is indicated to inhibit cell death signaling. *n* = 6–9 per group, *p ≤ 0.05, **p ≤ 0.01, ***p ≤ 0.001, ****p ≤ 0.0001 versus saline in the respective brain region (Multiple t-tests or Mann–Whitney)

**Fig. 6:**
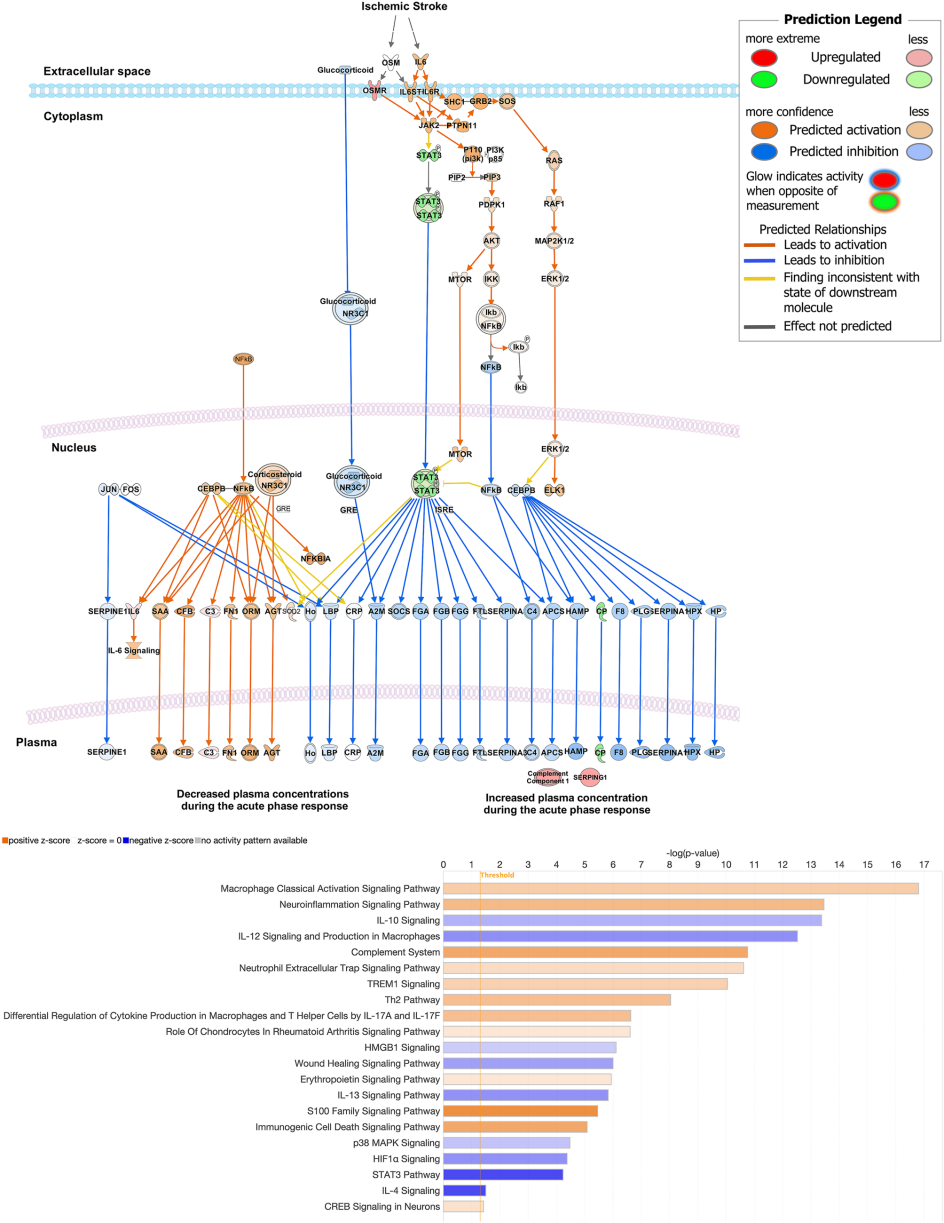
NPD1 + RvD1 inhibits downstream acute response signaling activated by MCAo and alters canonical pathways associated with stroke pathophysiology. Acute signaling pathway generated by IPA of predicted bioactivity from fold change ratio values of NPD1 + RvD1 treated ipsilesional penumbra samples

Fold change values (2^-∆∆Ct) uploaded to IPA for functional analysis displayed pathways in which the enriched genes regulate signaling either positively or negatively, with predicted inhibition or activation based on the fold change of the treated samples. The induction of gene expression reflecting neurotoxic astrocytes by activated microglia is inhibited by NPD1 + RvD1. Cell death-related differential gene expression changes indicate a potential downstream inhibition of apoptosis by NPD1 + RvD1 in the ipsilesional penumbra (Fig. 5a–b). Components of the acute signaling pathway were predicted to be both inhibited and activated (Fig. 6a). Functional analysis of canonical pathways through IPA found that expression of genes involved in macrophage activation, complement system, NETs, *Trem1, Th2, Il-17*, and CREB signaling pathways were activated by NPD1 + RvD1. Genes involved in key signaling processes after stroke, including *Il-10, Il-12, Hmgb1, Il-13, p38 MAPK, HIF1α, Stat3,* and* Il-4*, were inhibited by NPD1 + RvD1 (Fig. 6b).

## Discussion

This study is the first to assess effective doses and therapeutic windows for NPD1 + RvD1 treatment following MCAo in rats. We show that NPD1 + RvD1 remarkably improves neurological function and reduces lesion volume in acute ischemic stroke when administered promptly in moderate doses. We also demonstrated a broad therapeutic window of neuroprotection with moderate-dose (222 µg/kg) of NPD1 + RvD1, such that treatment initiated even 6 h after stroke onset is highly effective.

Various experimental therapies protect and recover neural tissues after ischemic stroke (Savitz et al. 2017). However, the persistence of long-term behavioral disabilities due to severe side effects and narrow therapeutic time windows is still a concern (Savitz et al. 2017). In the current study, we showed that NPD1 or RvD1 alone and in combination positively impacted the functional recovery of rats after seven days of MCAo induction. We selected an effective middle dose (222 µg/kg) for our therapeutic window study, which improved neurological scores and markedly reduced lesion volumes. The effect of NPD1 + RvD1 was demonstrated clearly in the ischemic core and penumbra when administered at 3, 4, 5, and 6 h and reduced T2WI total lesion volume by 95, 82, 81, and 84%, respectively, compared to the vehicle.

Combinatory therapy in treating ischemic stroke is an attractive approach due to the multifaceted nature of the I/R injury. Thus, a combination of neuroprotectants is being explored (Connell et al. 2014; Casas et al. 2019), including promoting neurological recovery by remodeling brain tissue (Hermann and Chopp 2012), in rats treated with retinoic acid in an environmental enrichment following transient MCAo, to stimulate neurogenesis in the subventricular zone and enhance neuronal survival (Rodriguez de Turco et al. 2002). Combined pharmacotherapy and behavioral manipulation enhance poststroke striatal neurogenesis and decrease infarct volume without detectable functional recovery (Rodriguez de Turco et al. 2002). This neurorestorative approach might not necessarily require a restricted therapeutic time window.

Another potential strategy is to use a neuroprotective agent combined with thrombolysis (Knecht et al. 2018; Jin et al. 2020; Blanco et al. 2022). t-PA is the only approved thrombolytic agent for acute ischemic stroke and protects the brain by recanalizing an occluded vessel and restoring blood flow to the ischemic brain, whereas neuroprotectants act directly upon the brain. Combination therapy could extend the current therapeutic time window of t-PA from 4.5 h and reduce adverse effects such as intracerebral hemorrhage (Jiang et al. 2015; Knecht et al. 2018). Combining t-PA treatment with neuroprotective drugs such as NMDA receptor antagonists (Lekieffre et al. 1997), free radical scavengers (Kimura et al. 2012), matrix metalloprotease inhibitors (Tan et al. 2015), and application of nanoparticle drug delivery systems (Fukuta et al. 2022) have synergistic effects in experimental studies. However, combination strategies have yet to be thoroughly examined clinically (Ishrat et al. 2019).

Functional deficits in rodents following MCAo resemble sensorimotor deficits. Focal ischemic stroke leads to impaired sensorimotor and cognitive functions, with 70–80% of patients displaying hemiparesis after stroke. Since the stroke therapy goal is restoring behavioral functions, two tests of the sensorimotor battery were used in the present study to detect neurological deficits following stroke. We show here that NPD1 and RvD1 improved overall neurological recovery, highlighted by the time course of recovery of postural reflex and proprioceptive and tactile contralateral forelimb reactions through the 7-day survival period.

We demonstrated that NPD1 + RvD1 mediates protection of the ipsilesional penumbra with increased expression of microglial and astrocyte genes involved in anti-inflammatory signaling and neuronal cell survival.

Reactive astrocytes activate a multitude of signaling pathways (Li et al. 2022) and, in ischemic stroke, exhibit molecular phenotypes that may be beneficial or protective (Zamanian et al. 2012). MCAo induces neuroprotective astrocyte reactivity displaying 150 reactive glial genes preferentially expressed and 57 genes expressed by LPS (Zamanian et al. 2012). Thus neuroinflammatory astrocytes involve NF-κB and JAK2/STAT3 signaling, increasing the expression of neurotrophic factors promoting neuronal survival and growth (Rakers et al. 2019).

Pan-reactive astrocyte genes (*Cxcl10* and *Osmr*), A1 neurotoxic genes (*Amigo2, Ugt1a, Gpc4, H2-D1,* and *Iigp1*), and A2 neuroprotective genes (*Ptx3, Thbs2,* and *Tm4sf1*) were all upregulated by NPD1 + RvD1 in the ipsilesional penumbra at 24 h after stroke. Pan-reactive *Osmr* regulates the function and survival of neurons recruiting *OSMRβ* during stroke (Guo et al. 2015). This activates JAK/STAT3 pro-survival signaling in neurons, and upregulation by NPD1 + RvD1 increasing *Osmr* expression provides a mechanism of penumbral protection. Additionally, the pan-reactive gene *Cxcl10*, which activates the *Cxcr3* receptor, is expressed by neurons and glia and can be either neuroprotective or detrimental in facilitating progression or resolution. Astrocytes release *Cxcl10*, which functions as a T lymphocyte chemoattractant, following ischemic injury and is also responsible for the recruitment of oligodendrocytes and remyelination (Cekanaviciute and Buckwalter 2016; Li et al. 2022).

*Amigo2* is an A1-associated gene that participates in the negative regulation of programmed cell death and as a positive regulator of synapse assembly (Laeremans et al. 2013). The observed increase in *Amigo2* may indicate NPD1 + RvD1 mediated synaptogenesis and cell death inhibition. Astrocytes induce excitatory synapse formation through the secretion of glypicans (*Gpc4*) and thrombospondins (*Thbs1* and *Thbs2*). Neurotoxic astrocytes cause neuronal damage through multiple mechanisms, one being reducing the secretion of *Gpc4* and *Thbs1/2* (Liddelow et al. 2017). *Tm4sf1*, an A2 marker involved in cell development regulation, activation, growth, and motility, is upregulated following ischemic stroke compared to sham astrocytes and further increased by NPD1 and RvD1 (Zamanian et al. 2012).

*H2-D1*, involved in antigen processing and presentation, participates in synapse alterations and reflects neurotoxic astrocyte phenotypes; it is increased by NPD1 and RvD1. However, the role of *H2-D1* in stroke pathogenesis is not clear but is associated with increased expression of pro-inflammatory genes (Mangold et al. 2017).

NPD1 + RvD1 mediated changes in homeostatic microglia genes (*Fcrls* and *Tmem119*), M1 genes (*Cd68, C3,* and *Msr1*), and M2 gene (*Cd163*) in the ipsilesional penumbra. FC receptor-like molecule (*Fcrls*) belongs to a family of receptors that bind IgG and identifies canonical microglia presenting on homeostatic phenotypes. Another indicator of non-reactive microglia is transmembrane 119 (*Tmem119*). NPD1 and RvD1 increased both *Fcrls* and *Tmem119*, increasing the quantity of homeostatic and non-reactive microglia following stroke onset (Young et al. 2021). Macrophage scavenger receptor 1 (*Msr1*), also named *Cd204*, contribute to I/R injury when overexpressed; however, loss of *Msr1* leads to reduced DAMP clearance resulting in severe inflammation, *Msr1* mediated clearance of DAMPs was observed to reduce infarct size in MCAo ameliorating neurologic deficits (Gudgeon et al. 2022).

*C3* is a central complement pathway protein that increases in the brain following ischemic stroke peaking 3 days after onset (Clarke et al. 2019; Ma et al. 2019b). *C3* modulates immune response and mediates downstream inflammation through interaction with the complement component 3a receptor (C3aR) (Clarke et al. 2019; Ma et al. 2019b). *C3* inhibition attenuates ischemic brain injury; however, conflicting studies show that *C3* deficiency impaired neurogenesis and implied neuroprotective bioactivity of *C3* in the sub-acute phase of stroke.

*CD68*, a marker of microglia phagocytic activity, peaks and invades the ischemic core at 7d after stroke onset (Perego et al. 2011). Hemoglobin scavenger receptor *Cd163* clears oxidative hemoglobin, leading to heme degradation by heme oxygenase-1 producing anti-inflammatory metabolites, and thus is an M2-phenotype-related marker. NPD1 and RvD1 increased the expression of *Cd163* in the ischemic penumbra 124-fold, demonstrating a mechanism through which inflammation is resolved (Jurga et al. 2020).

Neurotoxic astrocyte phenotypes are known to be induced through the release of *Tnf-a, IL-1a,* and *C1q* from activated microglia. In the ischemic penumbra, NPD1 + RvD1 decreased the expression of *Tnf-a* and *IL-1a,* which could indicate the inhibition of this cell–cell interaction to elicit neuroprotection (Liddelow et al. 2017). Cell death signaling pathway genes that promote cell survival (*Osmr, Amigo2,* and *Msr1*) were upregulated in the ipsilesional penumbra, while *Tgf-β* and *Tnf-a,* which promote cell death, were downregulated by NPD1 and RvD1.

The acute phase response encompasses multiple processes following the onset of stroke. The acute phase response pathway generated in IPA predicted inhibition and activation of components of this pathway based on the NPD1 + RvD1 mediated differential expression of genes in the ipsilesional penumbra. Canonical signaling pathways that exhibited the most significant degree of changes by NPD1 + RvD1 in the ipsilesional penumbra included decreased expression of genes involved in *IL-10, HMGB1, IL-13, p38 MAPK, HIF1α, STAT3,* and *IL-4* signaling; and increases in genes involved in macrophage activation, neuroinflammation, complement system, NETs, *Trem1*, *Th2, S100* family, and CREB signaling.

## Conclusion

We demonstrated that the lipid mediators NPD1+RvD1 provide additive neuroprotection in a rat model of ischemic stroke, increasing the therapeutic window and improving outcomes compared to monotherapy with either NPD1 or RvD1. This reveals a potential therapeutic avenue to be explored for ischemic stroke and neurological injuries. NPD1+RvD1 mediates protection of the ipsilesional penumbra with increased expression of microglial and astrocyte genes involved in anti-inflammatory signaling, synapse circuitry protection, and neuronal cell survival.

### Limitations of Research and Alternatives

Characterization of astrocyte and microglia cell types based on studies that define gene markers for individual phenotypes is not the most accurate way to characterize these cells. There is variability in literature identifying genes that determine the phenotypes, with many genes serving dual functions, potentially enhancing recovery in the acute phase of stroke based on their bioactivity. The mechanistic interpretation of the data is largely correlatory due to analysis of the gene set in IPA, which can only predict causal upstream relationships; this will necessitate investigation of additional genes involved in signaling mechanisms identified with this analysis in subsequent studies to determine causality. Additionally, investigation of each of the lipids' effect on gene expression independently is planned to determine if NPD1 and RvD1 have shared or differing mechanistic targets. A next step is to assess the proteins of each because posttranscriptional changes might modify the end products. Also, the inclusion of females to compare with males would be important following ischemic stroke, and the changes induced by NPD1 + RvD1 would provide important mechanistic information.

## Supplementary Information

Below is the link to the electronic supplementary material.

Supplementary file1 (DOCX 2015 KB)

Supplementary file2 (DOCX 127 KB)

## Data Availability

There are no restrictions on materials. All data are available in the main text or the supplementary materials.
