# Supplementary material for: NPD1 Plus RvD1 Mediated Ischemic Stroke Penumbra Protection Increases Expression of Pro-homeostatic Microglial and Astrocyte Genes
Source: Cell Mol Neurobiol. 2023 Jun 4;43(7):3555–73. doi: 10.1007/s10571-023-01363-3 (PMC10477115; doi:10.1007/s10571-023-01363-3)
Supplement: Supplementary file 1 — Supplementary file1 (DOCX 2015 KB) [file 10571_2023_1363_MOESM1_ESM.docx]

**NPD1 Plus RvD1 Mediated Ischemic Stroke Penumbra Protection Increases Expression of Pro-homeostatic Microglial and Astrocyte Genes**

*Cellular and Molecular Neurobiology*

Madigan M. Reid, Marie-Audrey I. Kautzmann, Gethein Andrew, Andre Obenaus, Pranab K. Mukherjee, Larissa Khoutorova, Jeff X. Ji, Cassia R. Roque, Reinaldo B. Oria, Bola F. Habeb, Ludmila Belayev, Nicolas G. Bazan

*Corresponding Authors: Nicolas G. Bazan and Ludmila Belayev

E-mail: nbazan@lsuhsc.edu and lbelay@lsuhsc.edu


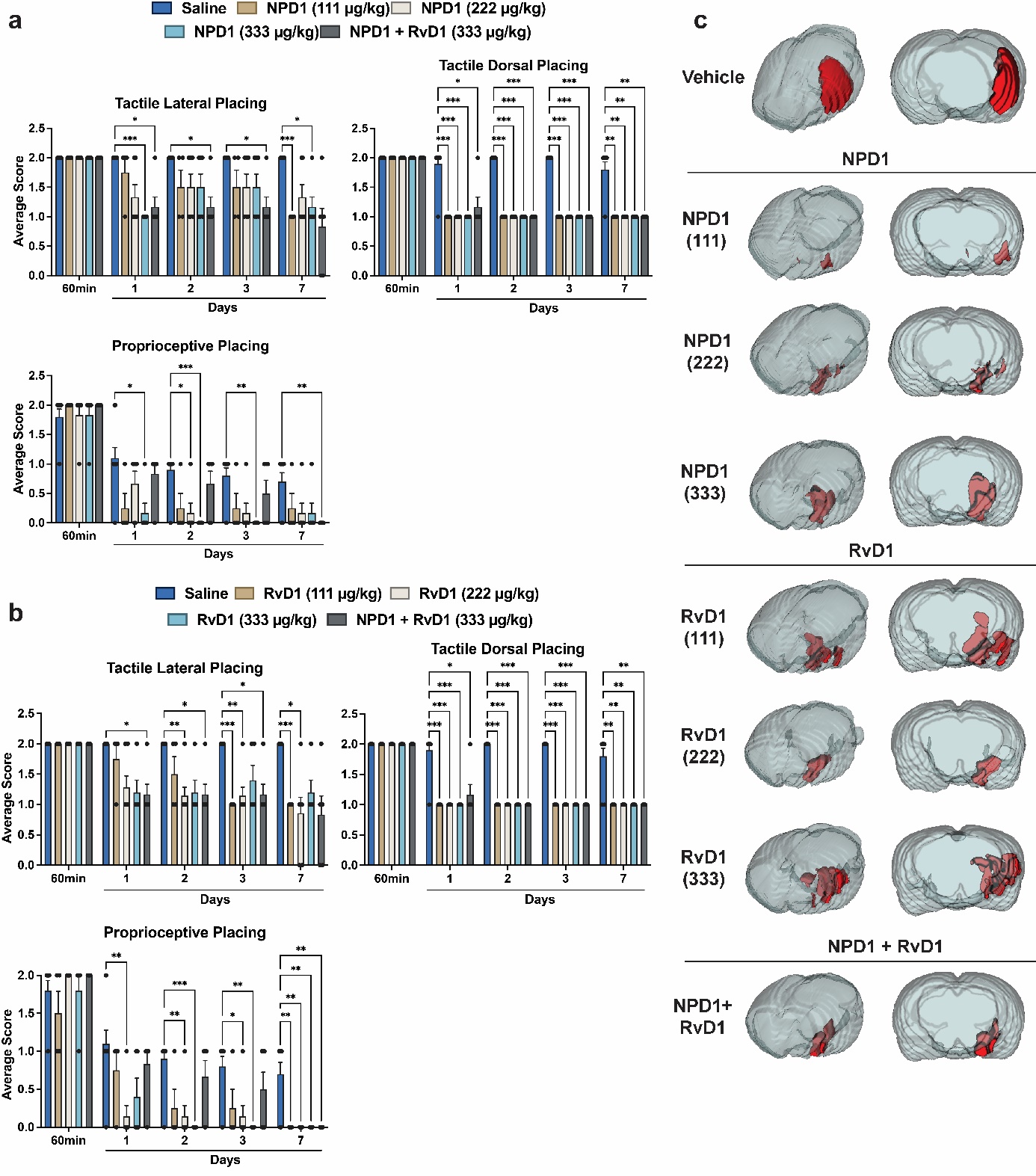


**Fig. S1. Time course of recovery of forelimb placing reactions to tactile (dorsal and lateral) and proprioceptive stimuli (normal = 0, maximal deficit = 2).** (**a-b**) All treatments improved tactile (lateral and dorsal) and proprioceptive placing at different times compared to the vehicle group. Values shown are means ± SEM; n= 8-6 per group, *p<0.05 versus saline group (Mann-Whitney test). (**c**) Three‑dimensional (3D) lesion volumes were computed from T2 maps from each group on day 7. A dramatic decrease in lesion volume was observed in all treated groups, primarily localized to small cortical and subcortical areas. The large lesion was observed in saline-treated rats in cortical and subcortical regions.

**
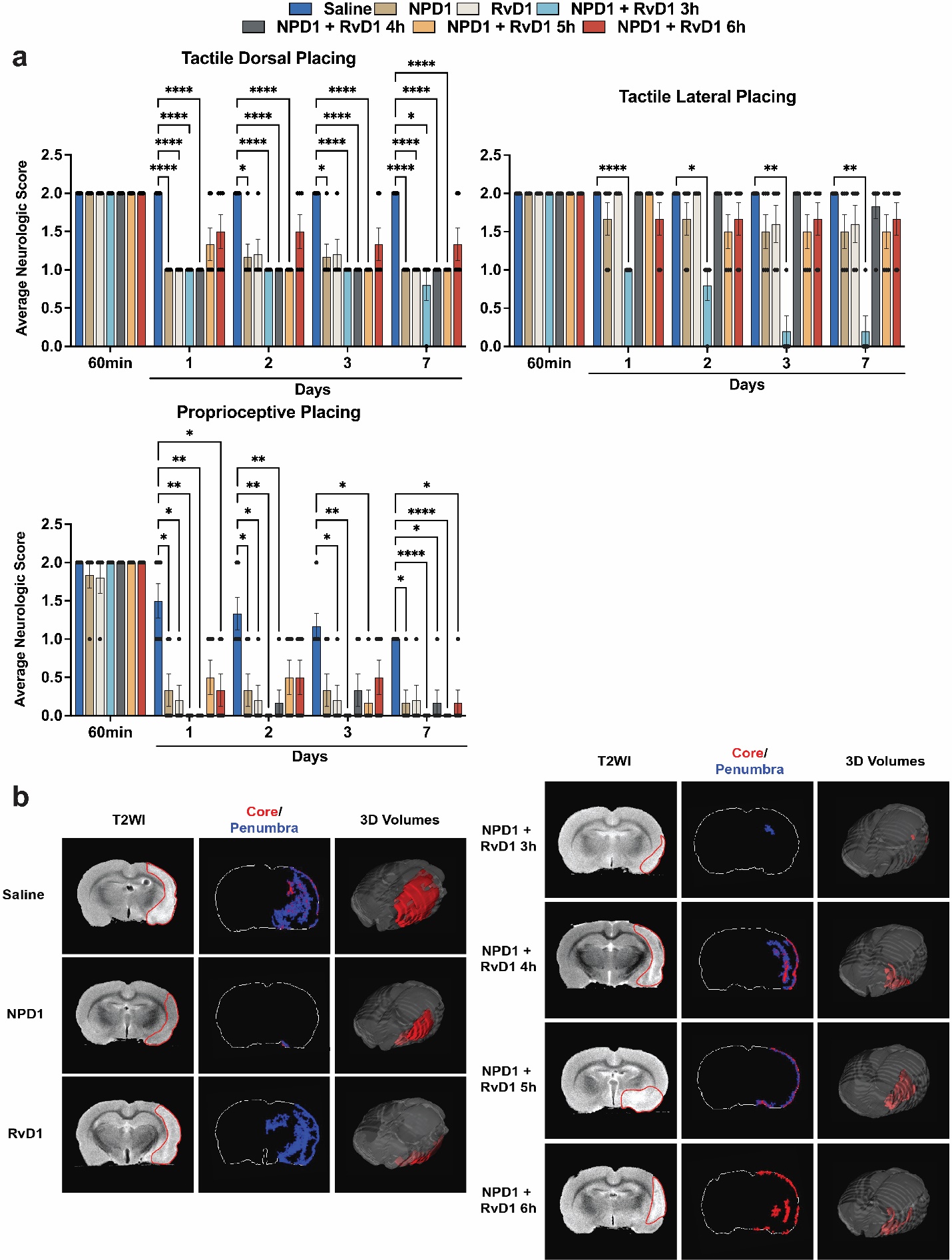
**

**Fig. S2. Time course of recovery of tactile (dorsal and lateral) and proprioceptive reactions (normal = 0, maximal deficit = 2) following MCAo.** (**a**) Treatment elicited improvement in placing reactions in all treated groups vs. saline-treated rats. Behavioral data are means ± SEM; n= 5-6 per group, *P<0.05 versus saline group (Mann-Whitney test). (**b**) Representative T2-weighted images (T2WI), core/penumbra, and three‑dimensional lesion volumes were computed from T2 maps from each group on day 7. Core (red) and penumbral (blue) tissues were automatically extracted from the entire brain using the MRI method of hierarchical region splitting for penumbra identification. T2 hyperintensities were observed in vehicle, NPD1, and RvD1-treated rats alone in the ischemic core and penumbra, consistent with edema formation. In contrast, all NPD1+RvD1 treated rats at 3, 4, 5, and 6 h had reduced lesion sizes in the core and penumbra.

**
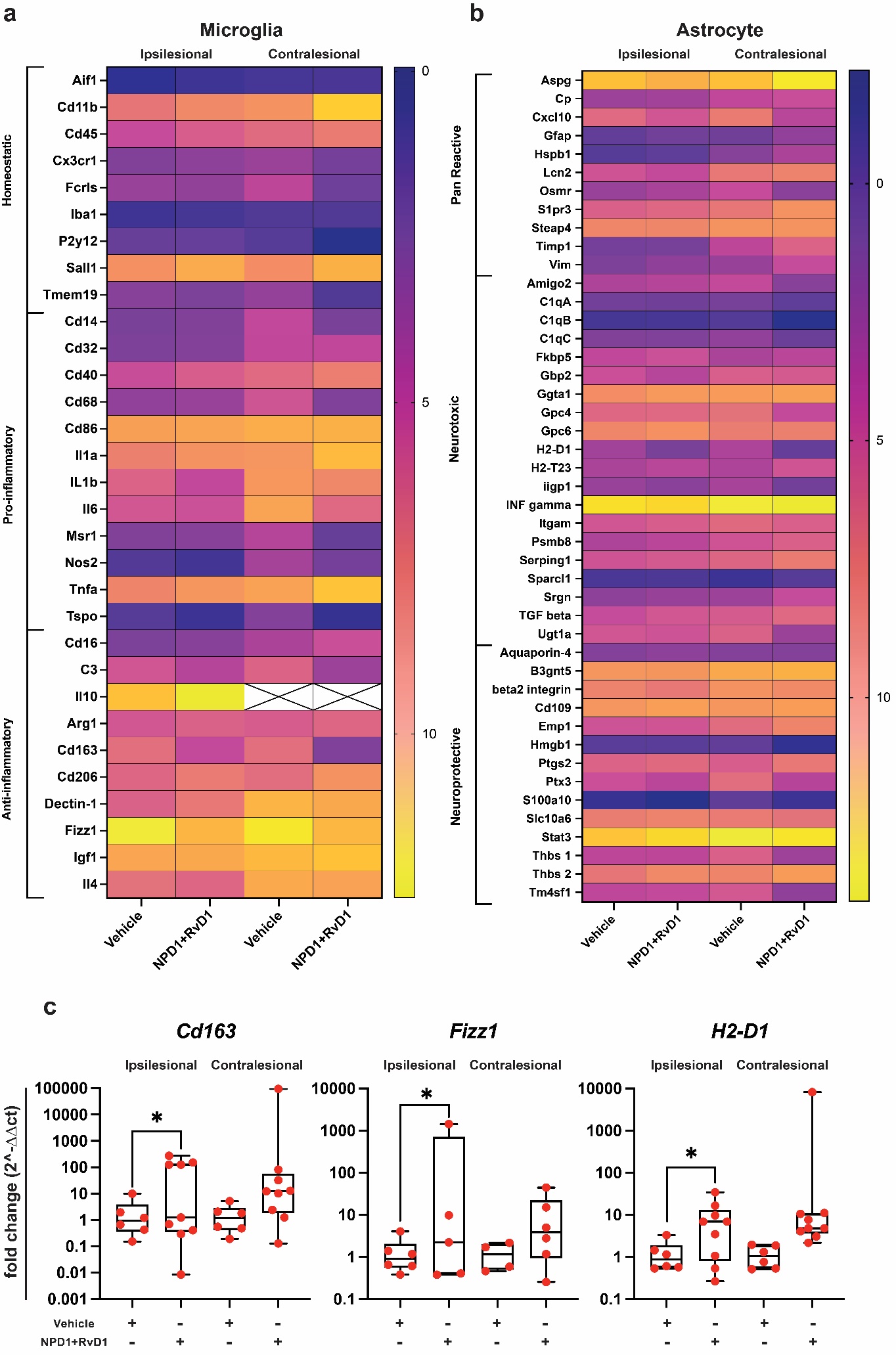
**

**Fig. S3. Ischemic core Expression of microglia and astrocyte genes at 24 h after MCAo.** (**a**) Heatmaps with mean ∆Ct values of astrocyte and microglia genes for control and NPD1+RvD1 ipsilesional and contralesional samples. (**b**) Boxplots of fold-change values for significantly changing genes. Values shown are means ± SEM; n= 6-9 per group, *p ≤0.05, **p≤0.01, ***p≤0.001, ****p≤0.0001 versus saline in the respective brain region (Multiple t-tests or Mann-Whitney).

**
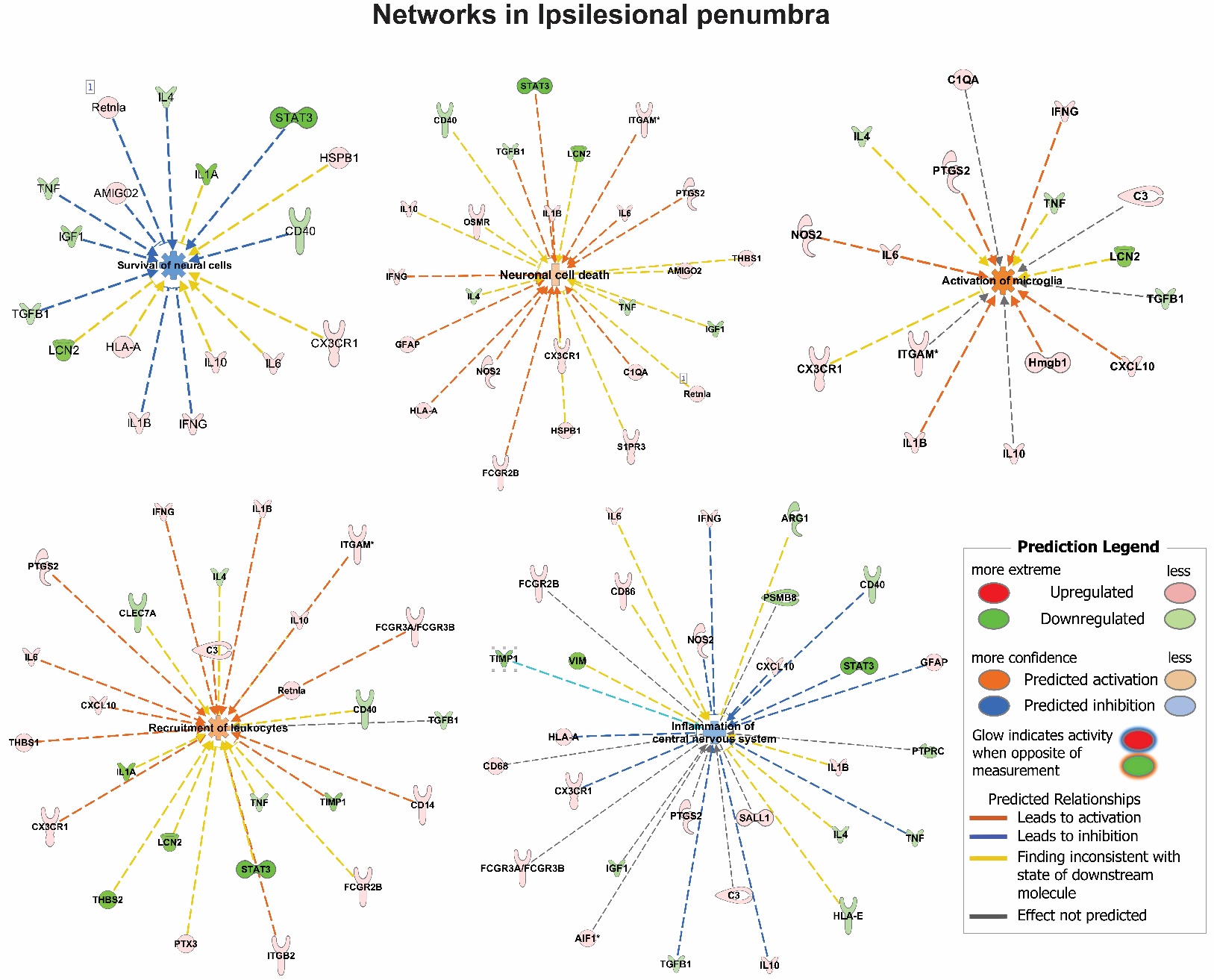
**

**Fig. S4. Network maps of predicted relationships between target genes and upstream or downstream regulation for microglia activation, leukocyte recruitment, neuronal survival, cell death, and inflammation of the central nervous system.** Green nodes indicate genes in which direct decreases in expression were measured, and red nodes indicate increased expression. Blue lines and nodes indicate predicted inhibition by NPD1+RvD1, and orange lines and nodes represent predicted activation.
