# Supplementary material for: NPD1 Plus RvD1 Mediated Ischemic Stroke Penumbra Protection Increases Expression of Pro-homeostatic Microglial and Astrocyte Genes
Source: Cell Mol Neurobiol. 2023 Jun 4;43(7):3555–73. doi: 10.1007/s10571-023-01363-3 (PMC10477115; doi:10.1007/s10571-023-01363-3)
Supplement: Supplementary file 2 — Supplementary file2 (DOCX 127 KB) [file 10571_2023_1363_MOESM2_ESM.docx]

**NPD1 Plus RvD1 Mediated Ischemic Stroke Penumbra Protection Increases Expression of Pro-homeostatic Microglial and Astrocyte Genes**

*Cellular and Molecular Neurobiology*

Madigan M. Reid, Marie-Audrey I. Kautzmann, Gethein Andrew, Andre Obenaus, Pranab K. Mukherjee, Larissa Khoutorova, Jeff X. Ji, Cassia R. Roque, Reinaldo B. Oria, Bola F. Habeb, Ludmila Belayev, Nicolas G. Bazan

*Corresponding Authors: Nicolas G. Bazan and Ludmila Belayev

E-mail: nbazan@lsuhsc.edu and lbelay@lsuhsc.edu

**Table S1. Primer Sequence and Ensembl ID Number.**

| Target or Reference Gene | Primer Sequence | Ensembl ID |
| --- | --- | --- |
| AIF1 | F- AAGGATTTGCAGGGAGGAAAAGC  R- CTCCATGTACTTCGTCTTGAAGG | ENSRNOG00000000853 |
| AMIGO2 | F – ACCGACGGCTGGCTAAGTAT  R - AGGGTGCATTCTGCCTTAACT | ENSRNOG00000007032 |
| AQUAPORIN-4 | F – GGAAGGCATGAGTGACGGA  R - GCTTGAGTCCAGACGCCTTT | ENSRNOG00000016043 |
| ARG1 | F -GCTTGAGTCCAGACGCCTTT  R -CTCCTCGAGGCTGTCCCTTA | ENSRNOG00000013304 |
| ASPG | F- GTACGGAAGGCCAGTTGGAA  R - GCCAAAGGTCTCCATGACCA | ENSRNOG00000012843 |
| B3GNT5 | F – AGTCTCCGAAGTCACACCCA  R - GCACGTCCAAATCCGTTGAC | ENSRNOG00000046258 |
| BETA2 INTEGRIN | F – CCTTCTCTCCACAGGACATGC  R - GTGAAGTTCAGCTTCTGGCAC | ENSRNOG00000001224 |
| C1QA | F – AGGTCACTTCATCTGTGCGG  R - CCCCTGCTAACACCTGGAAG | ENSRNOG00000012807 |
| C1QB | F – CCTTCTGCGACTATGCCCAA  R - CCTGCAGGTGAACAACCTCT | ENSRNOG00000012749 |
| C1QC | F – AGCATCAGTCGGTGTTCACG  R - GAGGATTGGTGATGGCGGAA | ENSRNOG00000012804 |
| C3 | F – CAAACTTCGGGGCAACAGTG  R - TCTTGCCCACAGGCAATAGG | ENSRNOG00000046834 |
| CD109 | F – AGGAATCATCAGACCCGGAGC  R - GCCGGAAGAACAAGAATTCCG | ENSRNOG00000025332 |
| CD11B | F – CTATTCGGCTCCAACCTGCT  R - TCGATCGTGTTGATGCTACCG | ENSRNOG00000019728 |
| CD14 | F – ATTGCCCAAGCACACTCACT  R - TCAGTCCTTTCTCGCCCAAC | ENSRNOG00000017819 |
| CD16 | F – CGTCCATGCAGACTGGCTAT  R - TTTCGCCAGCTATGGCATCT | ENSRNOG00000024382 |
| CD163 | F – TCCGGTTGAAGTTTTGTGACC  R - GTGGTCCCGATGACCGTATT | ENSRNOG00000010253 |
| CD206 | F – TGATTCCGGTCGCTGTTCAA  R - GAACGGAGATGGCGCTTAGA | ENSRNOG00000018251 |
| CD32 | F – AGGTTCCAGACACTCCTTCTG  R - CCGGAGCTTCAGGATGCTTG | ENSRNOG00000046452 |
| CD40 | F – AGGTGGTCAAGAAACCAAAGGA  R - GCAGTGTTGTGACCGGGATA | ENSRNOG00000018488 |
| CD45 | F – ACCATCCACTCTGGGCTTTG  R - ACGCACAGTAACGTTCCCAA | ENSRNOG00000000655 |
| CD68 | F – GCCCTCACCAAGTCCTAGTC  R - GCTTGGAGCTGAACACATGG | ENSRNOG00000037563 |
| CD86 | F – AGACATGTGTAACCTGCACCAT  R - GAGCTCACTCGGGCTTATGT | ENSRNOG00000038835 |
| CP | F – TTCATGCGGGGATGGTAACT  R - TGAACAGTTGTGTGGCTTTGA | ENSRNOG00000011913 |
| CX3CR1 | F – TCTTCACGTTCGGTCTGGTG  R - GGCAAAGTGGCCACAAAGAG | ENSRNOG00000018509 |
| CXCL10 | F – ACCCAGGGCCATAGGAAAAC  R - CTTTGGCTCACCGCTTTCAA | ENSRNOG00000022256 |
| DECTIN-1 | F – AGGAGAACCACAAACCCACAG  R - GCAAGGTCCAGAAAAGACGC | ENSRNOG00000054251 |
| EMP1 | F – CATCGCCACTGCCATTATGC  R - TGCAGTTCTTCCAAAGCCCT | ENSRNOG00000008676 |
| FCRLS | F – GTGCGTTTGGTGAATGGTCC  R - CTTCATGTCCCAGCCATCGT | ENSRNOG00000001955 |
| FIZZ1 | F – CAGCTGATGGTCCCAGTGAAT  R - GGAGGCCCATTTGCTCATAGA | ENSRNOG00000001955 |
| FKBP5 | F – CAGAGCAGGATGCCAAGGAA  R - TCCCATGGTCTGACTCTCGT | ENSRNOG00000022523 |
| GBP2 | F – TACCTGGAGCATTCGCTGAC  R - GTTGCTTCCTCAGAGCAGGT | ENSRNOG00000031743 |
| GFAP | F – GAAATTGCTGGAGGGCGAAG  R - ATTTGGTGTCCAGGCTGGTT | ENSRNOG00000002919 |
| GGTA1 | F – GCTGAAGACCTCACTCTCAGG  R - GAGTTCTATGGAGCTCCCGC | ENSRNOG00000019179 |
| GPC4 | F – ACTCTGCTCTCCTGCGACG  R - GGAAAAGGATCGGTGACCTCG | ENSRNOG00000002413 |
| GPC6 | F – ACACCCGCTTTCGACCCTA  R - CTGCATGGTCCTCCACACAGT | ENSRNOG00000046660 |
| H2-D1 | F – ATGGAACCTTCCAGAAGTGGG  R - GAAGTAAGTTGGAGTCGGTGGA | ENSRNOG00000030712 |
| H2-T23 | F – ATTGGAGCTGTTGTGAGGAGG  R - CCACGAGGCAACTGTCTTTTC | ENSRNOG00000000777 |
| HMGB1 | F – GCCCATTTTGGGTCACATGG  R - ACCCCTACCACAATATGCAGG | ENSRNOG00000058908 |
| HSPB1 | F – GTGGAGATCACTGGCAAGCA  R - ACCTGGAGGGAGCGTGTATT | ENSRNOG00000023546 |
| IBA1 | F – GCTATGAGCCAGAGCAAGGAT  R - GCTTGTTGATCCCATCCAACC | ENSRNOG00000000853 |
| IGF1 | F – GTACCAAAATGAGCGCACCTC  R - GCTGGTAAAGGTGAGCAAGC | ENSRNOG00000004517 |
| IIGP1 | F – ATTTGGCTCGAAGCCTTTGC  R - ACGGCATTTGCCAGTCCTTA | ENSRNOG00000038960 |
| IL10 | F – CCTCTGGATACAGCTGCGAC  R - TGGCCTTGTAGACACCTTTGT | ENSRNOG00000004647 |
| IL1A | F – CGCTTGAGTCGGCAAAGAAA  R - AGACAGATGGTCAATGGCAGA | ENSRNOG00000004575 |
| IL1B | F – CCTATGTCTTGCCCGTGGAG  R - CACACACTAGCAGGTCGTCA | ENSRNOG00000004649 |
| IL4 | F – CCCTGAACTCAACTGTGAAATAGCA  R - CCCAAGTCAAGGGCTTGGAA | ENSRNOG00000007624 |
| IL6 | F – TCTCCGCAAGAGACTTCCAG  R - AAGTCTCCTCTCCGGACTTGT | ENSRNOG00000010278 |
| INF GAMMA | F – CATCGCCAAGTTCGAGGTGA  R - CACCGACTCCTTTTCCGCTT | ENSRNOG00000007468 |
| ITGAM | F – GACTCCGCATTTGCCCTACT  R - TGCCCACAATGAGTGGTACAG | ENSRNOG00000019728 |
| LCN2 | F – ATTGTATGAACAGCGATGATGCAC  R - CCAGGTAGAAACGGAACTCCAGA | ENSRNOG00000013973 |
| MSR1 | F – TGGCTGAATGAAGTGATGTGC  R - CAAGTGACCCCAGCATCTTCT | ENSRNOG00000012779 |
| NOS2 | F – CAAGTGGCCGACACTGACTA  R - TTCAGTTCATCGGACAGCCC | ENSRNOG00000057443 |
| OSMR | F – TCCCTGGTCGAAAGTCCAAC  R - CATGGGTGGGTTCGTGACAA | ENSRNOG00000033192 |
| P2RY12 | F – TTGCACGGATTCCCTACACC  R - GGGTGCTCTCCTTCACGTAG | ENSRNOG00000013902 |
| PSMB8 | F – TGCAGCCTCCAAGCTACTTT  R - GCTGCCTGTGGAGAACATCT | ENSRNOG00000000456 |
| PTGS2 | F – CTCAGCCATGCAGCAAATCC  R - GGGTGGGCTTCAGCAGTAAT | ENSRNOG00000002525 |
| PTX3 | F – TATGGCACCAAGTGGAACCC  R - GGAGACCACAGTATCCGCAG | ENSRNOG00000012280 |
| S100A10 | F – AGGGAGTTCCCTGGGTTTTT  R - AAAGCTCTGGAAGCCCACTT | ENSRNOG00000023226 |
| SALL1 | F - CATTTCCAATCCGACCCCGA  R - CTTTGCTCTTAGTGGGGCGA | ENSRNOG00000013907 |
| SERPING1 | F – CAGAGGCTAACTGGCTTCGT  R - TGTGGCTGGTCACTTCAGAAT | ENSRNOG00000007457 |
| SLC10A6 | F – GCTGTGCATTGCCATGATGC  R - GCCTGATATGCTGCGACAATG | ENSRNOG00000002057 |
| S1PR3 | F – CTTGCAGAACGAGAGCCTGT  R - CCTCAACAGTCCACGAGAGG | ENSRNOG00000014524 |
| SPARCL1 | F – GCCGACACTGGAAGATGCTA  R - CTGTCGACTGTTCATGGGCT | ENSRNOG00000015093 |
| SRGN | F – AGAAAGGACCACGGTTCGAC  R - TGGGGAAGAAATCATTCGGGA | ENSRNOG00000000394 |
| STAT3 | F – GACCGCGTCGGCTAGGA  R - TGAGCCATCCTGCCGCAAT | ENSRNOG00000019742 |
| STEAP4 | F – GGCTCTCCAGTCAGGAACAC  R - AAGAGTGCGAGCAATGTCCA | ENSRNOG00000008602 |
| TGF BETA | F – CTGCTGACCCCCACTGATAC  R - AGCCCTGTATTCCGTCTCCT | ENSRNOG00000020652 |
| THBS1 | F – TAGCTGGAAATGTGGTGCGT  R - AGCAAGCATCAGGCACTTCT | ENSRNOG00000045829 |
| THBS2 | F – TGGAAGGACTACACCGCCTA  R - GAGTCAGCCATGACCTGCTT | ENSRNOG00000010529 |
| TIMP1 | F – CTGCAACTCGGACCTGGTTA  R - CAGCGTCGAATCCTTTGAGC | ENSRNOG00000010208 |
| TM4SF1 | F – TGCTTCCCAGACTTGGTTGT  R - TGAGAGAGGGTGTCACTTCAGA | ENSRNOG00000015812 |
| TMEM119 | F – GACTCCTCCCTGCTTGCTTTC  R - CCAAAACTTCGAGTCACTGCT | ENSRNOG00000000700 |
| TNFA | F – ATGGGCTCCCTCTCATCAGT  R - GCTTGGTGGTTTGCTACGAC | ENSRNOG00000070745 |
| TSPO | F – TGTGGATCTTTCCAGAACAGCA  R - CGCACAAAGTAGGCTCCCAT | ENSRNOG00000010549 |
| UGT1A | F – GGAAGCTGTTAGTGATCCCC  R - TGCTATGACCACCACTTCGT | ENSRNOG00000018740 |
| VIM | F – CATGCGGCTGCGAGAAAAAT  R - TTCAAGGTCAAGACGTGCCA | ENSRNOG00000018087 |
| ACTB | F – CCCGCGAGTACAACCTTCT  R - CGACGAGCGCAGCGATA | ENSRNOG00000034254 |
| GAPDH | F - GCATCTTCTTGTGCAGTGCC  R - GGTAACCAGGCGTCCGATAC | ENSRNOG00000018630 |
| GUSB | F – CAGTTGTGTGGGTGAATGGG  R - GGTCAGTGTGTTGTTGATGGC | ENSRNOG00000000913 |
| PGK1 | F – AAAGTCAGCCATGTGAGCACT  R - CCACTAGCTGCACTAACACC | ENSRNOG00000058249 |
| PP1A | F – GCAGACTTTGCTTTCCTTGG  R - CGAGAGGTCCTTTTCACCAG | ENSRNOG00000018708 |
| RPL13A | F – GGATCCCTCCACCCTATGACA  R - CTGGTACTTCCACCCGACCTC | ENSRNOG00000020618 |
| TFRC | F – CCGGCCTATATGCTTGGGTA  R - CAAGGGAGCACTCTGAAGCA | ENSRNOG00000001766 |

Primer table of all targets. Forward and reverse primer sequences and Ensembl gene ID are included.

**Table S2. Dose-Response Neurologic Statistics.**

| Total Neurologic Score | | |  |  |  |  |  |
| --- | --- | --- | --- | --- | --- | --- | --- |
|  | **Below threshold?** | **P value** | **Mean rank of Saline** | **Mean rank of NPD1 (111 µg/kg)** | **Mean rank diff.** | **Mann-Whitney U** | **Adjusted P Value** |
| 60min | No | >0.999999 | 7.3 | 8 | 0.7 | 18 | >0.999999 |
| 1 | Yes | 0.000999 | 9.5 | 2.5 | 7 | 0 | 0.004985 |
| 2 | Yes | 0.000999 | 9.5 | 2.5 | 7 | 0 | 0.004985 |
| 3 | Yes | 0.000999 | 9.5 | 2.5 | 7 | 0 | 0.004985 |
| 7 | Yes | 0.000999 | 9.5 | 2.5 | 7 | 0 | 0.004985 |
|  | **Below threshold?** | **P value** | **Mean rank of Saline** | **Mean rank of NPD1 (222 µg/kg)** | **Mean rank diff.** | **Mann-Whitney U** | **Adjusted P Value** |
| 60min | No | >0.999999 | 8.7 | 8.167 | 0.5333 | 28 | >0.999999 |
| 1 | Yes | 0.000125 | 11.5 | 3.5 | 8 | 0 | 0.000624 |
| 2 | Yes | 0.000125 | 11.5 | 3.5 | 8 | 0 | 0.000624 |
| 3 | Yes | 0.00025 | 11.5 | 3.5 | 8 | 0 | 0.000624 |
| 7 | Yes | 0.000125 | 11.5 | 3.5 | 8 | 0 | 0.000624 |
|  | **Below threshold?** | **P value** | **Mean rank of Saline** | **Mean rank of NPD1 (333 µg/kg)** | **Mean rank diff.** | **Mann-Whitney U** | **Adjusted P Value** |
| 60min | No | >0.999999 | 8.7 | 8.167 | 0.5333 | 28 | >0.999999 |
| 1 | Yes | 0.000125 | 11.5 | 3.5 | 8 | 0 | 0.000624 |
| 2 | Yes | 0.000125 | 11.5 | 3.5 | 8 | 0 | 0.000624 |
| 3 | Yes | 0.00025 | 11.5 | 3.5 | 8 | 0 | 0.000624 |
| 7 | Yes | 0.000125 | 11.5 | 3.5 | 8 | 0 | 0.000624 |
|  | **Below threshold?** | **P value** | **Mean rank of Saline** | **Mean rank of RvD1 (111 µg/kg)** | **Mean rank diff.** | **Mann-Whitney U** | **Adjusted P Value** |
| 60min | No | 0.175824 | 8.3 | 5.5 | 2.8 | 12 | 0.175824 |
| 1 | Yes | 0.003996 | 9.35 | 2.875 | 6.475 | 1.5 | 0.007976 |
| 2 | Yes | 0.000999 | 9.5 | 2.5 | 7 | 0 | 0.004985 |
| 3 | Yes | 0.000999 | 9.5 | 2.5 | 7 | 0 | 0.004985 |
| 7 | Yes | 0.000999 | 9.5 | 2.5 | 7 | 0 | 0.004985 |
|  | **Below threshold?** | **P value** | **Mean rank of Saline** | **Mean rank of RvD1 (222 µg/kg)** | **Mean rank diff.** | **Mann-Whitney U** | **Adjusted P Value** |
| 60min | No | >0.999999 | 8.65 | 9.5 | -0.85 | 31.5 | >0.999999 |
| 1 | Yes | 0.000257 | 12.35 | 4.214 | 8.136 | 1.5 | 0.000514 |
| 2 | Yes | 0.000051 | 12.5 | 4 | 8.5 | 0 | 0.000257 |
| 3 | Yes | 0.000051 | 12.5 | 4 | 8.5 | 0 | 0.000257 |
| 7 | Yes | 0.000051 | 12.5 | 4 | 8.5 | 0 | 0.000257 |
|  | **Below threshold?** | **P value** | **Mean rank of Saline** | **Mean rank of RvD1 (333 µg/kg)** | **Mean rank diff.** | **Mann-Whitney U** | **Adjusted P Value** |
| 60min | No | >0.999999 | 8.25 | 7.5 | 0.75 | 22.5 | >0.999999 |
| 1 | Yes | 0.001332 | 10.35 | 3.3 | 7.05 | 1.5 | 0.002662 |
| 2 | Yes | 0.000333 | 10.5 | 3 | 7.5 | 0 | 0.001664 |
| 3 | Yes | 0.000333 | 10.5 | 3 | 7.5 | 0 | 0.001664 |
| 7 | Yes | 0.000666 | 10.5 | 3 | 7.5 | 0 | 0.001997 |
|  | **Below threshold?** | **P value** | **Mean rank of Saline** | **Mean rank of NPD1 + RvD1** | **Mean rank diff.** | **Mann-Whitney U** | **Adjusted P Value** |
| 60min | No | >0.999999 | 8.2 | 9 | -0.8 | 27 | >0.999999 |
| 1 | Yes | 0.000125 | 11.5 | 3.5 | 8 | 0 | 0.000624 |
| 2 | Yes | 0.000125 | 11.5 | 3.5 | 8 | 0 | 0.000624 |
| 3 | Yes | 0.00025 | 11.5 | 3.5 | 8 | 0 | 0.000624 |
| 7 | Yes | 0.000125 | 11.5 | 3.5 | 8 | 0 | 0.000624 |

Statistics for dose-response neurologic assessment. Comparisons were made using nonparametric Mann-Whitney U test; all treatment groups were compared to Control (saline).

**Table S3. MRI Analysis Lesion Distribution Statistics.**

| Therapeutic-Window |  |  |  |  |  |  |  |  |  |  |  |  |  |  |  |  |  |
| --- | --- | --- | --- | --- | --- | --- | --- | --- | --- | --- | --- | --- | --- | --- | --- | --- | --- |
| Total | | | | | | **Penumbra** | | | | | | **Core** | | | | | |
| Dunnett's multiple comparisons test | Predicted (LS) mean diff. | 95.00% CI of diff. | Below threshold? | Summary | Adjusted P Value | **Dunnett's multiple comparisons test** | Predicted (LS) mean diff. | 95.00% CI of diff. | Below threshold? | Summary | Adjusted P Value | **Dunnett's multiple comparisons test** | Predicted (LS) mean diff. | 95.00% CI of diff. | Below threshold? | Summary | Adjusted P Value |
|  |  |  |  |  |  |  |  |  |  |  |  |  |  |  |  |  |  |
| 3.64 |  |  |  |  |  | **3.64** |  |  |  |  |  | **3.64** |  |  |  |  |  |
| Saline vs. NPD1 (222µg/kg) | 6.367 | 2.615 to 10.12 | Yes | *** | <.001 | **Saline vs. NPD1 (222µg/kg)** | 3.697 | 0.7030 to 6.690 | Yes | ** | 0.009 | **Saline vs. NPD1 (222µg/kg)** | 2.677 | 0.8571 to 4.496 | Yes | ** | 0.001 |
| Saline vs. RvD1 (222µg/kg) | 4.914 | 0.9910 to 8.837 | Yes | ** | 0.008 | **Saline vs. RvD1 (222µg/kg)** | 3.372 | 0.2408 to 6.503 | Yes | * | 0.03 | **Saline vs. RvD1 (222µg/kg)** | 1.549 | -0.3538 to 3.452 | No | ns | 0.149 |
| Saline vs. RvD1 + NPD1 3h | 6.803 | 3.051 to 10.55 | Yes | *** | <.001 | **Saline vs. RvD1 + NPD1 3h** | 4.155 | 1.161 to 7.148 | Yes | ** | 0.003 | **Saline vs. RvD1 + NPD1 3h** | 2.655 | 0.8351 to 4.474 | Yes | ** | 0.001 |
| Saline vs. RvD1 + NPD1 4h | 6.657 | 3.024 to 10.29 | Yes | *** | <.001 | **Saline vs. RvD1 + NPD1 4h** | 3.749 | 0.7550 to 6.742 | Yes | ** | 0.008 | **Saline vs. RvD1 + NPD1 4h** | 2.783 | 0.9631 to 4.602 | Yes | *** | <.001 |
| Saline vs. RvD1 + NPD1 5h | 6.223 | 2.471 to 9.974 | Yes | *** | <.001 | **Saline vs. RvD1 + NPD1 5h** | 3.809 | 0.8150 to 6.802 | Yes | ** | 0.007 | **Saline vs. RvD1 + NPD1 5h** | 2.421 | 0.6011 to 4.240 | Yes | ** | 0.004 |
| Saline vs. RvD1 + NPD1 6h | 7.317 | 3.565 to 11.07 | Yes | *** | <.001 | **Saline vs. RvD1 + NPD1 6h** | 4.457 | 1.463 to 7.450 | Yes | ** | 0.001 | **Saline vs. RvD1 + NPD1 6h** | 2.867 | 1.047 to 4.686 | Yes | *** | <.001 |
|  |  |  |  |  |  |  |  |  |  |  |  |  |  |  |  |  |  |
| 2.89 |  |  |  |  |  | **2.89** |  |  |  |  |  | **2.89** |  |  |  |  |  |
| Saline vs. NPD1 (222µg/kg) | 9.149 | 5.398 to 12.90 | Yes | *** | <.001 | **Saline vs. NPD1 (222µg/kg)** | 6.785 | 3.791 to 9.778 | Yes | *** | <.001 | **Saline vs. NPD1 (222µg/kg)** | 2.361 | 0.5417 to 4.181 | Yes | ** | 0.006 |
| Saline vs. RvD1 (222µg/kg) | 5.393 | 1.470 to 9.317 | Yes | ** | 0.003 | **Saline vs. RvD1 (222µg/kg)** | 5.182 | 2.051 to 8.313 | Yes | *** | <.001 | **Saline vs. RvD1 (222µg/kg)** | 0.2108 | -1.692 to 2.114 | No | ns | >.999 |
| Saline vs. RvD1 + NPD1 3h | 10.11 | 6.356 to 13.86 | Yes | *** | <.001 | **Saline vs. RvD1 + NPD1 3h** | 6.961 | 3.967 to 9.954 | Yes | *** | <.001 | **Saline vs. RvD1 + NPD1 3h** | 3.143 | 1.324 to 4.963 | Yes | *** | <.001 |
| Saline vs. RvD1 + NPD1 4h | 9.587 | 5.954 to 13.22 | Yes | *** | <.001 | **Saline vs. RvD1 + NPD1 4h** | 6.247 | 3.253 to 9.240 | Yes | *** | <.001 | **Saline vs. RvD1 + NPD1 4h** | 3.117 | 1.298 to 4.937 | Yes | *** | <.001 |
| Saline vs. RvD1 + NPD1 5h | 8.557 | 4.806 to 12.31 | Yes | *** | <.001 | **Saline vs. RvD1 + NPD1 5h** | 6.407 | 3.413 to 9.400 | Yes | *** | <.001 | **Saline vs. RvD1 + NPD1 5h** | 2.147 | 0.3277 to 3.967 | Yes | * | 0.014 |
| Saline vs. RvD1 + NPD1 6h | 10.63 | 6.878 to 14.38 | Yes | *** | <.001 | **Saline vs. RvD1 + NPD1 6h** | 7.537 | 4.543 to 10.53 | Yes | *** | <.001 | **Saline vs. RvD1 + NPD1 6h** | 3.089 | 1.270 to 4.909 | Yes | *** | <.001 |
|  |  |  |  |  |  |  |  |  |  |  |  |  |  |  |  |  |  |
| 2.14 |  |  |  |  |  | **2.14** |  |  |  |  |  | **2.14** |  |  |  |  |  |
| Saline vs. NPD1 (222µg/kg) | 10.2 | 6.445 to 13.95 | Yes | *** | <.001 | **Saline vs. NPD1 (222µg/kg)** | 7.37 | 4.376 to 10.36 | Yes | *** | <.001 | **Saline vs. NPD1 (222µg/kg)** | 2.826 | 1.006 to 4.646 | Yes | *** | <.001 |
| Saline vs. RvD1 (222µg/kg) | 8.385 | 4.462 to 12.31 | Yes | *** | <.001 | **Saline vs. RvD1 (222µg/kg)** | 6.18 | 3.049 to 9.311 | Yes | *** | <.001 | **Saline vs. RvD1 (222µg/kg)** | 2.205 | 0.3020 to 4.108 | Yes | * | 0.017 |
| Saline vs. RvD1 + NPD1 3h | 13.44 | 9.689 to 17.19 | Yes | *** | <.001 | **Saline vs. RvD1 + NPD1 3h** | 10.34 | 7.342 to 13.33 | Yes | *** | <.001 | **Saline vs. RvD1 + NPD1 3h** | 3.104 | 1.284 to 4.924 | Yes | *** | <.001 |
| Saline vs. RvD1 + NPD1 4h | 11.64 | 8.011 to 15.28 | Yes | *** | <.001 | **Saline vs. RvD1 + NPD1 4h** | 8.644 | 5.650 to 11.64 | Yes | *** | <.001 | **Saline vs. RvD1 + NPD1 4h** | 2.778 | 0.9584 to 4.598 | Yes | *** | <.001 |
| Saline vs. RvD1 + NPD1 5h | 11.74 | 7.987 to 15.49 | Yes | *** | <.001 | **Saline vs. RvD1 + NPD1 5h** | 9.63 | 6.636 to 12.62 | Yes | *** | <.001 | **Saline vs. RvD1 + NPD1 5h** | 2.106 | 0.2864 to 3.926 | Yes | * | 0.017 |
| Saline vs. RvD1 + NPD1 6h | 12.41 | 8.659 to 16.16 | Yes | *** | <.001 | **Saline vs. RvD1 + NPD1 6h** | 9.488 | 6.494 to 12.48 | Yes | *** | <.001 | **Saline vs. RvD1 + NPD1 6h** | 2.922 | 1.102 to 4.742 | Yes | *** | <.001 |
|  |  |  |  |  |  |  |  |  |  |  |  |  |  |  |  |  |  |
| 1.64 |  |  |  |  |  | **1.64** |  |  |  |  |  | **1.64** |  |  |  |  |  |
| Saline vs. NPD1 (222µg/kg) | 4.527 | 0.7760 to 8.279 | Yes | * | 0.012 | **Saline vs. NPD1 (222µg/kg)** | 2.864 | -0.1297 to 5.858 | No | ns | 0.066 | **Saline vs. NPD1 (222µg/kg)** | 1.663 | -0.1563 to 3.483 | No | ns | 0.085 |
| Saline vs. RvD1 (222µg/kg) | 2.573 | -1.350 to 6.497 | No | ns | 0.317 | **Saline vs. RvD1 (222µg/kg)** | 1.455 | -1.676 to 4.586 | No | ns | 0.64 | **Saline vs. RvD1 (222µg/kg)** | 1.118 | -0.7846 to 3.021 | No | ns | 0.421 |
| Saline vs. RvD1 + NPD1 3h | 8.929 | 5.178 to 12.68 | Yes | *** | <.001 | **Saline vs. RvD1 + NPD1 3h** | 6.376 | 3.382 to 9.370 | Yes | *** | <.001 | **Saline vs. RvD1 + NPD1 3h** | 2.553 | 0.7337 to 4.373 | Yes | ** | 0.002 |
| Saline vs. RvD1 + NPD1 4h | 7.198 | 3.566 to 10.83 | Yes | *** | <.001 | **Saline vs. RvD1 + NPD1 4h** | 5.256 | 2.262 to 8.250 | Yes | *** | <.001 | **Saline vs. RvD1 + NPD1 4h** | 2.281 | 0.4617 to 4.101 | Yes | ** | 0.008 |
| Saline vs. RvD1 + NPD1 5h | 7.657 | 3.906 to 11.41 | Yes | *** | <.001 | **Saline vs. RvD1 + NPD1 5h** | 5.65 | 2.656 to 8.644 | Yes | *** | <.001 | **Saline vs. RvD1 + NPD1 5h** | 2.005 | 0.1857 to 3.825 | Yes | * | 0.025 |
| Saline vs. RvD1 + NPD1 6h | 8.743 | 4.992 to 12.49 | Yes | *** | <.001 | **Saline vs. RvD1 + NPD1 6h** | 6.224 | 3.230 to 9.218 | Yes | *** | <.001 | **Saline vs. RvD1 + NPD1 6h** | 2.519 | 0.6997 to 4.339 | Yes | ** | 0.003 |
|  |  |  |  |  |  |  |  |  |  |  |  |  |  |  |  |  |  |
| 0.64 |  |  |  |  |  | **0.64** |  |  |  |  |  | **0.64** |  |  |  |  |  |
| Saline vs. NPD1 (222µg/kg) | 3.744 | -0.007294 to 7.495 | No | ns | 0.051 | **Saline vs. NPD1 (222µg/kg)** | 2.44 | -0.5537 to 5.434 | No | ns | 0.148 | **Saline vs. NPD1 (222µg/kg)** | 1.305 | -0.5143 to 3.125 | No | ns | 0.242 |
| Saline vs. RvD1 (222µg/kg) | 2.593 | -1.331 to 6.516 | No | ns | 0.31 | **Saline vs. RvD1 (222µg/kg)** | 0.1475 | -2.983 to 3.278 | No | ns | >.999 | **Saline vs. RvD1 (222µg/kg)** | 2.446 | 0.5429 to 4.349 | Yes | ** | 0.006 |
| Saline vs. RvD1 + NPD1 3h | 6.318 | 2.567 to 10.07 | Yes | *** | <.001 | **Saline vs. RvD1 + NPD1 3h** | 3.182 | 0.1883 to 6.176 | Yes | * | 0.033 | **Saline vs. RvD1 + NPD1 3h** | 3.139 | 1.320 to 4.959 | Yes | *** | <.001 |
| Saline vs. RvD1 + NPD1 4h | 4.913 | 1.281 to 8.546 | Yes | ** | 0.004 | **Saline vs. RvD1 + NPD1 4h** | 2.264 | -0.7297 to 5.258 | No | ns | 0.2 | **Saline vs. RvD1 + NPD1 4h** | 2.945 | 1.126 to 4.765 | Yes | *** | <.001 |
| Saline vs. RvD1 + NPD1 5h | 5.47 | 1.719 to 9.221 | Yes | ** | 0.001 | **Saline vs. RvD1 + NPD1 5h** | 2.79 | -0.2037 to 5.784 | No | ns | 0.076 | **Saline vs. RvD1 + NPD1 5h** | 2.685 | 0.8657 to 4.505 | Yes | ** | 0.001 |
| Saline vs. RvD1 + NPD1 6h | 5.954 | 2.203 to 9.705 | Yes | *** | <.001 | **Saline vs. RvD1 + NPD1 6h** | 2.89 | -0.1037 to 5.884 | No | ns | 0.062 | **Saline vs. RvD1 + NPD1 6h** | 3.065 | 1.246 to 4.885 | Yes | *** | <.001 |
|  |  |  |  |  |  |  |  |  |  |  |  |  |  |  |  |  |  |
| -1.11 |  |  |  |  |  | **-1.11** |  |  |  |  |  | **-1.11** |  |  |  |  |  |
| Saline vs. NPD1 (222µg/kg) | 4.908 | 1.157 to 8.659 | Yes | ** | 0.005 | **Saline vs. NPD1 (222µg/kg)** | 3.162 | 0.1683 to 6.156 | Yes | * | 0.034 | **Saline vs. NPD1 (222µg/kg)** | 1.744 | -0.07559 to 3.564 | No | ns | 0.065 |
| Saline vs. RvD1 (222µg/kg) | 1.683 | -2.241 to 5.606 | No | ns | 0.705 | **Saline vs. RvD1 (222µg/kg)** | 0.6 | -2.531 to 3.731 | No | ns | 0.989 | **Saline vs. RvD1 (222µg/kg)** | 1.083 | -0.8205 to 2.985 | No | ns | 0.452 |
| Saline vs. RvD1 + NPD1 3h | 6.12 | 2.369 to 9.871 | Yes | *** | <.001 | **Saline vs. RvD1 + NPD1 3h** | 4.08 | 1.086 to 7.074 | Yes | ** | 0.003 | **Saline vs. RvD1 + NPD1 3h** | 2.04 | 0.2204 to 3.860 | Yes | * | 0.022 |
| Saline vs. RvD1 + NPD1 4h | 4.965 | 1.333 to 8.597 | Yes | ** | 0.003 | **Saline vs. RvD1 + NPD1 4h** | 3.266 | 0.2723 to 6.260 | Yes | * | 0.027 | **Saline vs. RvD1 + NPD1 4h** | 1.468 | -0.3516 to 3.288 | No | ns | 0.155 |
| Saline vs. RvD1 + NPD1 5h | 4.646 | 0.8947 to 8.397 | Yes | ** | 0.009 | **Saline vs. RvD1 + NPD1 5h** | 2.818 | -0.1757 to 5.812 | No | ns | 0.072 | **Saline vs. RvD1 + NPD1 5h** | 1.826 | 0.006412 to 3.646 | Yes | * | 0.049 |
| Saline vs. RvD1 + NPD1 6h | 5.46 | 1.709 to 9.211 | Yes | ** | 0.001 | **Saline vs. RvD1 + NPD1 6h** | 3.634 | 0.6403 to 6.628 | Yes | * | 0.011 | **Saline vs. RvD1 + NPD1 6h** | 1.828 | 0.008412 to 3.648 | Yes | * | 0.049 |
|  |  |  |  |  |  |  |  |  |  |  |  |  |  |  |  |  |  |
| -1.61 |  |  |  |  |  | **-1.61** |  |  |  |  |  | **-1.61** |  |  |  |  |  |
| Saline vs. NPD1 (222µg/kg) | 1.075 | -2.676 to 4.827 | No | ns | 0.925 | **Saline vs. NPD1 (222µg/kg)** | 0.6067 | -2.387 to 3.600 | No | ns | 0.985 | **Saline vs. NPD1 (222µg/kg)** | 0.4647 | -1.355 to 2.284 | No | ns | 0.955 |
| Saline vs. RvD1 (222µg/kg) | 0.8108 | -3.112 to 4.734 | No | ns | 0.983 | **Saline vs. RvD1 (222µg/kg)** | 0.7842 | -2.347 to 3.915 | No | ns | 0.959 | **Saline vs. RvD1 (222µg/kg)** | 0.02667 | -1.876 to 1.930 | No | ns | >.999 |
| Saline vs. RvD1 + NPD1 3h | 2.233 | -1.518 to 5.985 | No | ns | 0.407 | **Saline vs. RvD1 + NPD1 3h** | 1.477 | -1.517 to 4.470 | No | ns | 0.587 | **Saline vs. RvD1 + NPD1 3h** | 0.7567 | -1.063 to 2.576 | No | ns | 0.731 |
| Saline vs. RvD1 + NPD1 4h | 1.26 | -2.372 to 4.892 | No | ns | 0.846 | **Saline vs. RvD1 + NPD1 4h** | 1.293 | -1.701 to 4.286 | No | ns | 0.702 | **Saline vs. RvD1 + NPD1 4h** | 0.6707 | -1.149 to 2.490 | No | ns | 0.814 |
| Saline vs. RvD1 + NPD1 5h | 2.127 | -1.624 to 5.879 | No | ns | 0.453 | **Saline vs. RvD1 + NPD1 5h** | 1.459 | -1.535 to 4.452 | No | ns | 0.598 | **Saline vs. RvD1 + NPD1 5h** | 0.6687 | -1.151 to 2.488 | No | ns | 0.816 |
| Saline vs. RvD1 + NPD1 6h | 1.135 | -2.616 to 4.887 | No | ns | 0.907 | **Saline vs. RvD1 + NPD1 6h** | 1.381 | -1.613 to 4.374 | No | ns | 0.647 | **Saline vs. RvD1 + NPD1 6h** | -0.2433 | -2.063 to 1.576 | No | ns | 0.998 |
|  |  |  |  |  |  |  |  |  |  |  |  |  |  |  |  |  |  |
| -2.61 |  |  |  |  |  | **-2.61** |  |  |  |  |  | **-2.61** |  |  |  |  |  |
| Saline vs. NPD1 (222µg/kg) | 0.6673 | -3.084 to 4.419 | No | ns | 0.992 | **Saline vs. NPD1 (222µg/kg)** | 0.076 | -2.918 to 3.070 | No | ns | >.999 | **Saline vs. NPD1 (222µg/kg)** | 0.5913 | -1.228 to 2.411 | No | ns | 0.88 |
| Saline vs. RvD1 (222µg/kg) | 0.1933 | -3.730 to 4.117 | No | ns | >.999 | **Saline vs. RvD1 (222µg/kg)** | 0.19 | -2.941 to 3.321 | No | ns | >.999 | **Saline vs. RvD1 (222µg/kg)** | 0.003333 | -1.900 to 1.906 | No | ns | >.999 |
| Saline vs. RvD1 + NPD1 3h | 1.233 | -2.518 to 4.985 | No | ns | 0.873 | **Saline vs. RvD1 + NPD1 3h** | 0.19 | -2.804 to 3.184 | No | ns | >.999 | **Saline vs. RvD1 + NPD1 3h** | 1.043 | -0.7763 to 2.863 | No | ns | 0.444 |
| Saline vs. RvD1 + NPD1 4h | 1.162 | -2.471 to 4.794 | No | ns | 0.885 | **Saline vs. RvD1 + NPD1 4h** | 0.112 | -2.882 to 3.106 | No | ns | >.999 | **Saline vs. RvD1 + NPD1 4h** | 1.035 | -0.7843 to 2.855 | No | ns | 0.451 |
| Saline vs. RvD1 + NPD1 5h | 1.233 | -2.518 to 4.985 | No | ns | 0.873 | **Saline vs. RvD1 + NPD1 5h** | 0.19 | -2.804 to 3.184 | No | ns | >.999 | **Saline vs. RvD1 + NPD1 5h** | 1.043 | -0.7763 to 2.863 | No | ns | 0.444 |
| Saline vs. RvD1 + NPD1 6h | 0.9473 | -2.804 to 4.699 | No | ns | 0.956 | **Saline vs. RvD1 + NPD1 6h** | 0.19 | -2.804 to 3.184 | No | ns | >.999 | **Saline vs. RvD1 + NPD1 6h** | 0.7573 | -1.062 to 2.577 | No | ns | 0.731 |
|  |  |  |  |  |  |  |  |  |  |  |  |  |  |  |  |  |  |
| -3.61 |  |  |  |  |  | **-3.61** |  |  |  |  |  | **-3.61** |  |  |  |  |  |
| Saline vs. NPD1 (222µg/kg) | 0.1293 | -3.622 to 3.881 | No | ns | >.999 | **Saline vs. NPD1 (222µg/kg)** | 0.02 | -2.974 to 3.014 | No | ns | >.999 | **Saline vs. NPD1 (222µg/kg)** | 0.1093 | -1.710 to 1.929 | No | ns | >.999 |
| Saline vs. RvD1 (222µg/kg) | -0.1567 | -4.080 to 3.767 | No | ns | >.999 | **Saline vs. RvD1 (222µg/kg)** | 0.05 | -3.081 to 3.181 | No | ns | >.999 | **Saline vs. RvD1 (222µg/kg)** | -0.2067 | -2.110 to 1.696 | No | ns | >.999 |
| Saline vs. RvD1 + NPD1 3h | 0.3233 | -3.428 to 4.075 | No | ns | >.999 | **Saline vs. RvD1 + NPD1 3h** | 0.05 | -2.944 to 3.044 | No | ns | >.999 | **Saline vs. RvD1 + NPD1 3h** | 0.2733 | -1.546 to 2.093 | No | ns | 0.996 |
| Saline vs. RvD1 + NPD1 4h | 0.3233 | -3.309 to 3.956 | No | ns | >.999 | **Saline vs. RvD1 + NPD1 4h** | 0.05 | -2.944 to 3.044 | No | ns | >.999 | **Saline vs. RvD1 + NPD1 4h** | 0.2733 | -1.546 to 2.093 | No | ns | 0.996 |
| Saline vs. RvD1 + NPD1 5h | 0.3233 | -3.428 to 4.075 | No | ns | >.999 | **Saline vs. RvD1 + NPD1 5h** | 0.05 | -2.944 to 3.044 | No | ns | >.999 | **Saline vs. RvD1 + NPD1 5h** | 0.2733 | -1.546 to 2.093 | No | ns | 0.996 |
| Saline vs. RvD1 + NPD1 6h | -1.375 | -5.126 to 2.377 | No | ns | 0.815 | **Saline vs. RvD1 + NPD1 6h** | -0.432 | -3.426 to 2.562 | No | ns | 0.997 | **Saline vs. RvD1 + NPD1 6h** | -0.9447 | -2.764 to 0.8749 | No | ns | 0.539 |

Statistical significance of lesion area bregma level distribution in therapeutic-window study. All significant lesion areas compared to vehicle at the indicated bregma level are represented by an asterisk. Comparisons between groups were performed by Dunnets multiple comparison test *p<0.05, **p<0.01, ***p<0.001.

**Table S4. Therapeutic Window Neurologic Statistics.**

| Total Neurologic Score | | |  |  |  |  |  |
| --- | --- | --- | --- | --- | --- | --- | --- |
|  | **Below threshold?** | **P value** | **Mean rank of Saline** | **Mean rank of NPD1** | **Mean rank diff.** | **Mann-Whitney U** | **Adjusted P Value** |
| 60 min | No | 0.375 | 9 | 7.667 | 1.333 | 25 | 0.375 |
| 1 | Yes | 0.000125 | 11.5 | 3.5 | 8 | 0 | 0.000624 |
| 2 | Yes | 0.000125 | 11.5 | 3.5 | 8 | 0 | 0.000624 |
| 3 | Yes | 0.000125 | 11.5 | 3.5 | 8 | 0 | 0.000624 |
| 7 | Yes | 0.000125 | 11.5 | 3.5 | 8 | 0 | 0.000624 |
|  | **Below threshold?** | **P value** | **Mean rank of Saline** | **Mean rank of RvD1** | **Mean rank diff.** | **Mann-Whitney U** | **Adjusted P Value** |
| 60 min | No | >0.999999 | 8 | 8 | 0 | 25 | >0.999999 |
| 1 | Yes | 0.000666 | 10.5 | 3 | 7.5 | 0 | 0.001664 |
| 2 | Yes | 0.000333 | 10.5 | 3 | 7.5 | 0 | 0.001664 |
| 3 | Yes | 0.000333 | 10.5 | 3 | 7.5 | 0 | 0.001664 |
| 7 | Yes | 0.000333 | 10.5 | 3 | 7.5 | 0 | 0.001664 |
|  | **Below threshold?** | **P value** | **Mean rank of Saline** | **Mean rank of NPD1 + RvD1 3h** | **Mean rank diff.** | **Mann-Whitney U** | **Adjusted P Value** |
| 60 min | No | >0.999999 | 8 | 8 | 0 | 25 | >0.999999 |
| 1 | Yes | 0.000666 | 10.5 | 3 | 7.5 | 0 | 0.001664 |
| 2 | Yes | 0.000333 | 10.5 | 3 | 7.5 | 0 | 0.001664 |
| 3 | Yes | 0.000333 | 10.5 | 3 | 7.5 | 0 | 0.001664 |
| 7 | Yes | 0.000333 | 10.5 | 3 | 7.5 | 0 | 0.001664 |
|  | **Below threshold?** | **P value** | **Mean rank of Saline** | **Mean rank of NPD1 + RvD1 4h** | **Mean rank diff.** | **Mann-Whitney U** | **Adjusted P Value** |
| 60 min | No | >0.999999 | 9 | 9 | 0 | 35 | >0.999999 |
| 1 | Yes | 0.000051 | 12.5 | 4 | 8.5 | 0 | 0.000257 |
| 2 | Yes | 0.000051 | 12.5 | 4 | 8.5 | 0 | 0.000257 |
| 3 | Yes | 0.000051 | 12.5 | 4 | 8.5 | 0 | 0.000257 |
| 7 | Yes | 0.000051 | 12.5 | 4 | 8.5 | 0 | 0.000257 |
|  | **Below threshold?** | **P value** | **Mean rank of Saline** | **Mean rank of NPD1 + RvD1 5h** | **Mean rank diff.** | **Mann-Whitney U** | **Adjusted P Value** |
| 60 min | No | >0.999999 | 8.5 | 8.5 | 0 | 30 | >0.999999 |
| 1 | Yes | 0.000125 | 11.5 | 3.5 | 8 | 0 | 0.000624 |
| 2 | Yes | 0.0005 | 11.35 | 3.75 | 7.6 | 1.5 | 0.000999 |
| 3 | Yes | 0.000125 | 11.5 | 3.5 | 8 | 0 | 0.000624 |
| 7 | Yes | 0.000125 | 11.5 | 3.5 | 8 | 0 | 0.000624 |
|  | **Below threshold?** | **P value** | **Mean rank of Saline** | **Mean rank of NPD1 + RvD1 6h** | **Mean rank diff.** | **Mann-Whitney U** | **Adjusted P Value** |
| 60 min | No | >0.999999 | 9 | 9 | 0 | 35 | >0.999999 |
| 1 | Yes | 0.000051 | 12.5 | 4 | 8.5 | 0 | 0.000257 |
| 2 | Yes | 0.000206 | 12.35 | 4.214 | 8.136 | 1.5 | 0.000463 |
| 3 | Yes | 0.000154 | 12.4 | 4.143 | 8.257 | 1 | 0.000463 |
| 7 | Yes | 0.000051 | 12.5 | 4 | 8.5 | 0 | 0.000257 |

Statistics for Therapeutic window neurologic assessment. Comparisons were made using nonparametric Mann-Whitney U test all treatment groups were compared to Control (saline).

**Table S5. MRI Analysis Lesion Distribution Statistics.**

| Dose-Response |  |  |  |  |  |  |  |  |  |  |  |  |  |  |  |  |  |
| --- | --- | --- | --- | --- | --- | --- | --- | --- | --- | --- | --- | --- | --- | --- | --- | --- | --- |
| Total | | | | | | **Penumbra** | | | | | | **Core** | | | | | |
| Dunnett's multiple comparisons test | Predicted (LS) mean diff. | 95.00% CI of diff. | Below threshold? | Summary | Adjusted P Value | **Dunnett's multiple comparisons test** | Predicted (LS) mean diff. | 95.00% CI of diff. | Below threshold? | Summary | Adjusted P Value | **Dunnett's multiple comparisons test** | Predicted (LS) mean diff. | 95.00% CI of diff. | Below threshold? | Summary | Adjusted P Value |
|  |  |  |  |  |  |  |  |  |  |  |  |  |  |  |  |  |  |
| 3.64 |  |  |  |  |  | **3.64** |  |  |  |  |  | **3.64** |  |  |  |  |  |
| Saline vs. NPD1 (111µg/kg) | 3.04 | -3.389 to 9.469 | No | ns | 0.7293 | **Saline vs. NPD1 (111µg/kg)** | 1.107 | -1.608 to 3.822 | No | ns | 0.842 | **Saline vs. NPD1 (111µg/kg)** | 1.932 | -2.624 to 6.487 | No | ns | 0.816 |
| Saline vs. NPD1 (222µg/kg) | 3.257 | -2.612 to 9.126 | No | ns | 0.5679 | **Saline vs. NPD1 (222µg/kg)** | 1.223 | -1.255 to 3.702 | No | ns | 0.69 | **Saline vs. NPD1 (222µg/kg)** | 2.032 | -2.127 to 6.190 | No | ns | 0.699 |
| Saline vs. NPD1 (333µg/kg) | 3.257 | -3.172 to 9.686 | No | ns | 0.664 | **Saline vs. NPD1 (333µg/kg)** | 1.223 | -1.492 to 3.938 | No | ns | 0.77 | **Saline vs. NPD1 (333µg/kg)** | 2.032 | -2.524 to 6.587 | No | ns | 0.779 |
| Saline vs. RvD1 (111µg/kg) | 3.257 | -3.172 to 9.686 | No | ns | 0.664 | **Saline vs. RvD1 (111µg/kg)** | 1.223 | -1.492 to 3.938 | No | ns | 0.77 | **Saline vs. RvD1 (111µg/kg)** | 2.032 | -2.524 to 6.587 | No | ns | 0.779 |
| Saline vs. RvD1 (222µg/kg) | 3.257 | -1.993 to 8.506 | No | ns | 0.4419 | **Saline vs. RvD1 (222µg/kg)** | 1.223 | -0.9936 to 3.440 | No | ns | 0.574 | **Saline vs. RvD1 (222µg/kg)** | 2.032 | -1.688 to 5.751 | No | ns | 0.585 |
| Saline vs. RvD1 (333µg/kg) | 0.2392 | -5.630 to 6.108 | No | ns | 0.9999 | **Saline vs. RvD1 (333µg/kg)** | -0.8842 | -3.363 to 1.594 | No | ns | 0.91 | **Saline vs. RvD1 (333µg/kg)** | 1.119 | -3.039 to 5.278 | No | ns | 0.978 |
| Saline vs. RvD1 + NPD1 | 1.005 | -4.501 to 6.510 | No | ns | 0.9973 | **Saline vs. RvD1 + NPD1 (333μg/kg)** | -0.1267 | -2.452 to 2.198 | No | ns | >.999 | **Saline vs. RvD1 + NPD1 (333μg/kg)** | 1.13 | -2.771 to 5.031 | No | ns | 0.968 |
|  |  |  |  |  |  |  |  |  |  |  |  |  |  |  |  |  |  |
| 2.89 |  |  |  |  |  | **2.89** |  |  |  |  |  | **2.89** |  |  |  |  |  |
| Saline vs. NPD1 (111µg/kg) | 5.303 | -1.126 to 11.73 | No | ns | 0.1573 | **Saline vs. NPD1 (111µg/kg)** | 1.62 | -1.095 to 4.335 | No | ns | 0.486 | **Saline vs. NPD1 (111µg/kg)** | 3.682 | -0.8738 to 8.237 | No | ns | 0.173 |
| Saline vs. NPD1 (222µg/kg) | 5.743 | -0.1256 to 11.61 | No | ns | 0.0582 | **Saline vs. NPD1 (222µg/kg)** | 1.963 | -0.5152 to 4.442 | No | ns | 0.19 | **Saline vs. NPD1 (222µg/kg)** | 3.778 | -0.3802 to 7.937 | No | ns | 0.094 |
| Saline vs. NPD1 (333µg/kg) | 5.743 | -0.6858 to 12.17 | No | ns | 0.1034 | **Saline vs. NPD1 (333µg/kg)** | 1.963 | -0.7518 to 4.678 | No | ns | 0.275 | **Saline vs. NPD1 (333µg/kg)** | 3.778 | -0.7772 to 8.334 | No | ns | 0.153 |
| Saline vs. RvD1 (111µg/kg) | 5.743 | -0.6858 to 12.17 | No | ns | 0.1034 | **Saline vs. RvD1 (111µg/kg)** | 1.963 | -0.7518 to 4.678 | No | ns | 0.275 | **Saline vs. RvD1 (111µg/kg)** | 3.778 | -0.7772 to 8.334 | No | ns | 0.153 |
| Saline vs. RvD1 (222µg/kg) | 5.122 | -0.1277 to 10.37 | No | ns | 0.0594 | **Saline vs. RvD1 (222µg/kg)** | 1.785 | -0.4319 to 4.002 | No | ns | 0.176 | **Saline vs. RvD1 (222µg/kg)** | 3.337 | -0.3829 to 7.056 | No | ns | 0.101 |
| Saline vs. RvD1 (333µg/kg) | 3.026 | -2.843 to 8.895 | No | ns | 0.6462 | **Saline vs. RvD1 (333µg/kg)** | 0.6008 | -1.878 to 3.079 | No | ns | 0.988 | **Saline vs. RvD1 (333µg/kg)** | 2.423 | -1.735 to 6.582 | No | ns | 0.513 |
| Saline vs. RvD1 + NPD1 | 3.185 | -2.320 to 8.691 | No | ns | 0.5212 | **Saline vs. RvD1 + NPD1 (333μg/kg)** | 1.173 | -1.152 to 3.498 | No | ns | 0.668 | **Saline vs. RvD1 + NPD1 (333μg/kg)** | 2.01 | -1.891 to 5.911 | No | ns | 0.647 |
|  |  |  |  |  |  |  |  |  |  |  |  |  |  |  |  |  |  |
| 2.14 |  |  |  |  |  | **2.14** |  |  |  |  |  | **2.14** |  |  |  |  |  |
| Saline vs. NPD1 (111µg/kg) | 9.798 | 3.369 to 16.23 | Yes | *** | 0.0005 | **Saline vs. NPD1 (111µg/kg)** | 3.805 | 1.090 to 6.520 | Yes | ** | 0.002 | **Saline vs. NPD1 (111µg/kg)** | 5.992 | 1.436 to 10.55 | Yes | ** | 0.004 |
| Saline vs. NPD1 (222µg/kg) | 9.137 | 3.268 to 15.01 | Yes | *** | 0.0003 | **Saline vs. NPD1 (222µg/kg)** | 3.596 | 1.117 to 6.074 | Yes | ** | 0.001 | **Saline vs. NPD1 (222µg/kg)** | 5.542 | 1.383 to 9.700 | Yes | ** | 0.003 |
| Saline vs. NPD1 (333µg/kg) | 9.732 | 3.303 to 16.16 | Yes | *** | 0.0005 | **Saline vs. NPD1 (333µg/kg)** | 3.738 | 1.023 to 6.453 | Yes | ** | 0.002 | **Saline vs. NPD1 (333µg/kg)** | 5.992 | 1.436 to 10.55 | Yes | ** | 0.004 |
| Saline vs. RvD1 (111µg/kg) | 7.205 | 0.7759 to 13.63 | Yes | * | 0.0199 | **Saline vs. RvD1 (111µg/kg)** | 1.812 | -0.9035 to 4.527 | No | ns | 0.36 | **Saline vs. RvD1 (111µg/kg)** | 5.392 | 0.8362 to 9.947 | Yes | * | 0.012 |
| Saline vs. RvD1 (222µg/kg) | 8.162 | 2.912 to 13.41 | Yes | *** | 0.0003 | **Saline vs. RvD1 (222µg/kg)** | 3.05 | 0.8331 to 5.267 | Yes | ** | 0.002 | **Saline vs. RvD1 (222µg/kg)** | 5.108 | 1.389 to 8.828 | Yes | ** | 0.002 |
| Saline vs. RvD1 (333µg/kg) | 7.944 | 2.075 to 13.81 | Yes | ** | 0.0026 | **Saline vs. RvD1 (333µg/kg)** | 2.766 | 0.2873 to 5.244 | Yes | * | 0.021 | **Saline vs. RvD1 (333µg/kg)** | 5.177 | 1.018 to 9.335 | Yes | ** | 0.007 |
| Saline vs. RvD1 + NPD1 | 6.2 | 0.6941 to 11.71 | Yes | * | 0.0191 | **Saline vs. RvD1 + NPD1 (333μg/kg)** | 2.548 | 0.2232 to 4.873 | Yes | * | 0.024 | **Saline vs. RvD1 + NPD1 (333μg/kg)** | 3.65 | -0.2514 to 7.551 | No | ns | 0.078 |
|  |  |  |  |  |  |  |  |  |  |  |  |  |  |  |  |  |  |
| 1.64 |  |  |  |  |  | **1.64** |  |  |  |  |  | **1.64** |  |  |  |  |  |
| Saline vs. NPD1 (111µg/kg) | 11.56 | 5.133 to 17.99 | Yes | **** | <0.0001 | **Saline vs. NPD1 (111µg/kg)** | 4.248 | 1.533 to 6.963 | Yes | *** | <.001 | **Saline vs. NPD1 (111µg/kg)** | 7.315 | 2.760 to 11.87 | Yes | *** | <.001 |
| Saline vs. NPD1 (222µg/kg) | 10.61 | 4.743 to 16.48 | Yes | **** | <0.0001 | **Saline vs. NPD1 (222µg/kg)** | 3.73 | 1.251 to 6.209 | Yes | *** | <.001 | **Saline vs. NPD1 (222µg/kg)** | 6.881 | 2.722 to 11.04 | Yes | *** | <.001 |
| Saline vs. NPD1 (333µg/kg) | 9.958 | 3.529 to 16.39 | Yes | *** | 0.0004 | **Saline vs. NPD1 (333µg/kg)** | 3.228 | 0.5132 to 5.943 | Yes | * | 0.011 | **Saline vs. NPD1 (333µg/kg)** | 6.732 | 2.176 to 11.29 | Yes | *** | <.001 |
| Saline vs. RvD1 (111µg/kg) | 10.12 | 3.689 to 16.55 | Yes | *** | 0.0003 | **Saline vs. RvD1 (111µg/kg)** | 3.545 | 0.8299 to 6.260 | Yes | ** | 0.004 | **Saline vs. RvD1 (111µg/kg)** | 6.575 | 2.020 to 11.13 | Yes | ** | 0.001 |
| Saline vs. RvD1 (222µg/kg) | 10.22 | 4.971 to 15.47 | Yes | **** | <0.0001 | **Saline vs. RvD1 (222µg/kg)** | 3.488 | 1.271 to 5.705 | Yes | *** | <.001 | **Saline vs. RvD1 (222µg/kg)** | 6.733 | 3.014 to 10.45 | Yes | *** | <.001 |
| Saline vs. RvD1 (333µg/kg) | 10.99 | 5.120 to 16.86 | Yes | **** | <0.0001 | **Saline vs. RvD1 (333µg/kg)** | 3.865 | 1.386 to 6.344 | Yes | *** | <.001 | **Saline vs. RvD1 (333µg/kg)** | 7.128 | 2.970 to 11.29 | Yes | *** | <.001 |
| Saline vs. RvD1 + NPD1 | 10.46 | 4.952 to 15.96 | Yes | **** | <0.0001 | **Saline vs. RvD1 + NPD1 (333μg/kg)** | 3.915 | 1.590 to 6.240 | Yes | *** | <.001 | **Saline vs. RvD1 + NPD1 (333μg/kg)** | 6.542 | 2.641 to 10.44 | Yes | *** | <.001 |
|  |  |  |  |  |  |  |  |  |  |  |  |  |  |  |  |  |  |
| 0.64 |  |  |  |  |  | **0.64** |  |  |  |  |  | **0.64** |  |  |  |  |  |
| Saline vs. NPD1 (111µg/kg) | 10.72 | 4.288 to 17.15 | Yes | **** | <0.0001 | **Saline vs. NPD1 (111µg/kg)** | 3.582 | 0.8665 to 6.297 | Yes | ** | 0.004 | **Saline vs. NPD1 (111µg/kg)** | 7.135 | 2.580 to 11.69 | Yes | *** | <.001 |
| Saline vs. NPD1 (222µg/kg) | 9.134 | 3.265 to 15.00 | Yes | *** | 0.0003 | **Saline vs. NPD1 (222µg/kg)** | 2.999 | 0.5206 to 5.478 | Yes | ** | 0.009 | **Saline vs. NPD1 (222µg/kg)** | 6.135 | 1.976 to 10.29 | Yes | *** | <.001 |
| Saline vs. NPD1 (333µg/kg) | 5.71 | -0.7191 to 12.14 | No | ns | 0.1068 | **Saline vs. NPD1 (333µg/kg)** | 2.005 | -0.7101 to 4.720 | No | ns | 0.254 | **Saline vs. NPD1 (333µg/kg)** | 3.705 | -0.8505 to 8.260 | No | ns | 0.168 |
| Saline vs. RvD1 (111µg/kg) | 6.183 | -0.2458 to 12.61 | No | ns | 0.0655 | **Saline vs. RvD1 (111µg/kg)** | 0.985 | -1.730 to 3.700 | No | ns | 0.903 | **Saline vs. RvD1 (111µg/kg)** | 5.198 | 0.6428 to 9.754 | Yes | * | 0.017 |
| Saline vs. RvD1 (222µg/kg) | 8.137 | 2.887 to 13.39 | Yes | *** | 0.0004 | **Saline vs. RvD1 (222µg/kg)** | 2.638 | 0.4214 to 4.855 | Yes | * | 0.011 | **Saline vs. RvD1 (222µg/kg)** | 5.498 | 1.779 to 9.218 | Yes | *** | <.001 |
| Saline vs. RvD1 (333µg/kg) | 10.72 | 4.848 to 16.59 | Yes | **** | <0.0001 | **Saline vs. RvD1 (333µg/kg)** | 3.582 | 1.103 to 6.060 | Yes | ** | 0.001 | **Saline vs. RvD1 (333µg/kg)** | 7.135 | 2.976 to 11.29 | Yes | *** | <.001 |
| Saline vs. RvD1 + NPD1 | 9.719 | 4.213 to 15.22 | Yes | **** | <0.0001 | **Saline vs. RvD1 + NPD1 (333μg/kg)** | 3.094 | 0.7686 to 5.419 | Yes | ** | 0.003 | **Saline vs. RvD1 + NPD1 (333μg/kg)** | 6.623 | 2.722 to 10.52 | Yes | *** | <.001 |
|  |  |  |  |  |  |  |  |  |  |  |  |  |  |  |  |  |  |
| -1.11 |  |  |  |  |  | **-1.11** |  |  |  |  |  | **-1.11** |  |  |  |  |  |
| Saline vs. NPD1 (111µg/kg) | 10.21 | 3.781 to 16.64 | Yes | *** | 0.0002 | **Saline vs. NPD1 (111µg/kg)** | 3.428 | 0.7132 to 6.143 | Yes | ** | 0.006 | **Saline vs. NPD1 (111µg/kg)** | 6.782 | 2.226 to 11.34 | Yes | *** | <.001 |
| Saline vs. NPD1 (222µg/kg) | 8.768 | 2.899 to 14.64 | Yes | *** | 0.0006 | **Saline vs. NPD1 (222µg/kg)** | 2.743 | 0.2648 to 5.222 | Yes | * | 0.022 | **Saline vs. NPD1 (222µg/kg)** | 6.022 | 1.863 to 10.18 | Yes | ** | 0.001 |
| Saline vs. NPD1 (333µg/kg) | 1.253 | -5.176 to 7.682 | No | ns | 0.9957 | **Saline vs. NPD1 (333µg/kg)** | 0.705 | -2.010 to 3.420 | No | ns | 0.982 | **Saline vs. NPD1 (333µg/kg)** | 0.545 | -4.010 to 5.100 | No | ns | >.999 |
| Saline vs. RvD1 (111µg/kg) | 9.54 | 3.111 to 15.97 | Yes | *** | 0.0007 | **Saline vs. RvD1 (111µg/kg)** | 2.935 | 0.2199 to 5.650 | Yes | * | 0.027 | **Saline vs. RvD1 (111µg/kg)** | 6.608 | 2.053 to 11.16 | Yes | ** | 0.001 |
| Saline vs. RvD1 (222µg/kg) | 9.11 | 3.861 to 14.36 | Yes | **** | <0.0001 | **Saline vs. RvD1 (222µg/kg)** | 3.013 | 0.7964 to 5.230 | Yes | ** | 0.002 | **Saline vs. RvD1 (222µg/kg)** | 6.098 | 2.379 to 9.818 | Yes | *** | <.001 |
| Saline vs. RvD1 (333µg/kg) | 9.343 | 3.474 to 15.21 | Yes | *** | 0.0002 | **Saline vs. RvD1 (333µg/kg)** | 3.348 | 0.8698 to 5.827 | Yes | ** | 0.003 | **Saline vs. RvD1 (333µg/kg)** | 5.994 | 1.836 to 10.15 | Yes | ** | 0.001 |
| Saline vs. RvD1 + NPD1 | 8.25 | 2.744 to 13.76 | Yes | *** | 0.0006 | **Saline vs. RvD1 + NPD1 (333μg/kg)** | 2.966 | 0.6412 to 5.291 | Yes | ** | 0.005 | **Saline vs. RvD1 + NPD1 (333μg/kg)** | 5.286 | 1.385 to 9.187 | Yes | ** | 0.003 |
|  |  |  |  |  |  |  |  |  |  |  |  |  |  |  |  |  |  |
| -1.61 |  |  |  |  |  | **-1.61** |  |  |  |  |  | **-1.61** |  |  |  |  |  |
| Saline vs. NPD1 (111µg/kg) | 4.338 | -2.091 to 10.77 | No | ns | 0.3477 | **Saline vs. NPD1 (111µg/kg)** | 2.07 | -0.6451 to 4.785 | No | ns | 0.224 | **Saline vs. NPD1 (111µg/kg)** | 2.267 | -2.289 to 6.822 | No | ns | 0.682 |
| Saline vs. NPD1 (222µg/kg) | 4.273 | -1.596 to 10.14 | No | ns | 0.268 | **Saline vs. NPD1 (222µg/kg)** | 1.888 | -0.5902 to 4.367 | No | ns | 0.224 | **Saline vs. NPD1 (222µg/kg)** | 2.386 | -1.773 to 6.544 | No | ns | 0.531 |
| Saline vs. NPD1 (333µg/kg) | 0.9583 | -5.471 to 7.387 | No | ns | 0.9994 | **Saline vs. NPD1 (333µg/kg)** | 1.067 | -1.648 to 3.782 | No | ns | 0.864 | **Saline vs. NPD1 (333µg/kg)** | -0.1133 | -4.669 to 4.442 | No | ns | >.999 |
| Saline vs. RvD1 (111µg/kg) | 5.008 | -1.421 to 11.44 | No | ns | 0.2044 | **Saline vs. RvD1 (111µg/kg)** | 2.273 | -0.4418 to 4.988 | No | ns | 0.146 | **Saline vs. RvD1 (111µg/kg)** | 2.733 | -1.822 to 7.289 | No | ns | 0.48 |
| Saline vs. RvD1 (222µg/kg) | 3.735 | -1.514 to 8.984 | No | ns | 0.2913 | **Saline vs. RvD1 (222µg/kg)** | 1.575 | -0.6419 to 3.792 | No | ns | 0.293 | **Saline vs. RvD1 (222µg/kg)** | 2.16 | -1.560 to 5.880 | No | ns | 0.517 |
| Saline vs. RvD1 (333µg/kg) | 4.896 | -0.9731 to 10.76 | No | ns | 0.1489 | **Saline vs. RvD1 (333µg/kg)** | 2.216 | -0.2627 to 4.694 | No | ns | 0.103 | **Saline vs. RvD1 (333µg/kg)** | 2.681 | -1.478 to 6.839 | No | ns | 0.398 |
| Saline vs. RvD1 + NPD1 | 2.628 | -2.877 to 8.134 | No | ns | 0.7207 | **Saline vs. RvD1 + NPD1 (333μg/kg)** | 1.217 | -1.108 to 3.542 | No | ns | 0.63 | **Saline vs. RvD1 + NPD1 (333μg/kg)** | 1.409 | -2.492 to 5.310 | No | ns | 0.905 |
|  |  |  |  |  |  |  |  |  |  |  |  |  |  |  |  |  |  |
| -2.61 |  |  |  |  |  | **-2.61** |  |  |  |  |  | **-2.61** |  |  |  |  |  |
| Saline vs. NPD1 (111µg/kg) | 1.443 | -4.986 to 7.872 | No | ns | 0.9922 | **Saline vs. NPD1 (111µg/kg)** | 0.56 | -2.155 to 3.275 | No | ns | 0.994 | **Saline vs. NPD1 (111µg/kg)** | 0.8817 | -3.674 to 5.437 | No | ns | 0.996 |
| Saline vs. NPD1 (222µg/kg) | 1.298 | -4.571 to 7.167 | No | ns | 0.9928 | **Saline vs. NPD1 (222µg/kg)** | 0.4475 | -2.031 to 2.926 | No | ns | 0.997 | **Saline vs. NPD1 (222µg/kg)** | 0.8467 | -3.312 to 5.005 | No | ns | 0.995 |
| Saline vs. NPD1 (333µg/kg) | 1.033 | -5.396 to 7.462 | No | ns | 0.9979 | **Saline vs. NPD1 (333µg/kg)** | 0.3767 | -2.338 to 3.092 | No | ns | >.999 | **Saline vs. NPD1 (333µg/kg)** | 0.655 | -3.900 to 5.210 | No | ns | >.999 |
| Saline vs. RvD1 (111µg/kg) | 1.243 | -5.186 to 7.672 | No | ns | 0.9961 | **Saline vs. RvD1 (111µg/kg)** | 0.5267 | -2.188 to 3.242 | No | ns | 0.996 | **Saline vs. RvD1 (111µg/kg)** | 0.7117 | -3.844 to 5.267 | No | ns | >.999 |
| Saline vs. RvD1 (222µg/kg) | 0.3917 | -4.858 to 5.641 | No | ns | 0.9997 | **Saline vs. RvD1 (222µg/kg)** | 0.2767 | -1.940 to 2.494 | No | ns | >.999 | **Saline vs. RvD1 (222µg/kg)** | 0.1133 | -3.606 to 3.833 | No | ns | >.999 |
| Saline vs. RvD1 (333µg/kg) | 0.8333 | -5.036 to 6.702 | No | ns | 0.9995 | **Saline vs. RvD1 (333µg/kg)** | 0.56 | -1.919 to 3.039 | No | ns | 0.992 | **Saline vs. RvD1 (333µg/kg)** | 0.2717 | -3.887 to 4.430 | No | ns | >.999 |
| Saline vs. RvD1 + NPD1 | -0.06867 | -5.574 to 5.437 | No | ns | >0.9999 | **Saline vs. RvD1 + NPD1 (333μg/kg)** | -0.102 | -2.427 to 2.223 | No | ns | >.999 | **Saline vs. RvD1 + NPD1 (333μg/kg)** | 0.03167 | -3.869 to 3.933 | No | ns | >.999 |
|  |  |  |  |  |  |  |  |  |  |  |  |  |  |  |  |  |  |
| -3.61 |  |  |  |  |  | **-3.61** |  |  |  |  |  | **-3.61** |  |  |  |  |  |
| Saline vs. NPD1 (111µg/kg) | 0.26 | -6.169 to 6.689 | No | ns | 0.9999 | **Saline vs. NPD1 (111µg/kg)** | 0.15 | -2.565 to 2.865 | No | ns | >.999 | **Saline vs. NPD1 (111µg/kg)** | 0.11 | -4.445 to 4.665 | No | ns | >.999 |
| Saline vs. NPD1 (222µg/kg) | 0.26 | -5.609 to 6.129 | No | ns | 0.9999 | **Saline vs. NPD1 (222µg/kg)** | 0.15 | -2.329 to 2.629 | No | ns | >.999 | **Saline vs. NPD1 (222µg/kg)** | 0.11 | -4.049 to 4.269 | No | ns | >.999 |
| Saline vs. NPD1 (333µg/kg) | -2.16 | -8.589 to 4.269 | No | ns | 0.932 | **Saline vs. NPD1 (333µg/kg)** | 0.02 | -2.695 to 2.735 | No | ns | >.999 | **Saline vs. NPD1 (333µg/kg)** | -2.18 | -6.735 to 2.375 | No | ns | 0.718 |
| Saline vs. RvD1 (111µg/kg) | -0.04667 | -6.476 to 6.382 | No | ns | >0.9999 | **Saline vs. RvD1 (111µg/kg)** | 0.01 | -2.705 to 2.725 | No | ns | >.999 | **Saline vs. RvD1 (111µg/kg)** | -0.05333 | -4.609 to 4.502 | No | ns | >.999 |
| Saline vs. RvD1 (222µg/kg) | 0.1867 | -5.063 to 5.436 | No | ns | 0.9999 | **Saline vs. RvD1 (222µg/kg)** | 0.1083 | -2.109 to 2.325 | No | ns | >.999 | **Saline vs. RvD1 (222µg/kg)** | 0.07833 | -3.641 to 3.798 | No | ns | >.999 |
| Saline vs. RvD1 (333µg/kg) | -0.1375 | -6.006 to 5.731 | No | ns | >0.9999 | **Saline vs. RvD1 (333µg/kg)** | -0.1325 | -2.611 to 2.346 | No | ns | >.999 | **Saline vs. RvD1 (333µg/kg)** | -0.005 | -4.164 to 4.154 | No | ns | >.999 |
| Saline vs. RvD1 + NPD1 | -0.664 | -6.170 to 4.842 | No | ns | 0.9996 | **Saline vs. RvD1 + NPD1 (333μg/kg)** | -0.244 | -2.569 to 2.081 | No | ns | >.999 | **Saline vs. RvD1 + NPD1 (333μg/kg)** | -0.42 | -4.321 to 3.481 | No | ns | >.999 |

Statistical significance of lesion area bregma level distribution in the dose-response study. All significant lesion areas compared to vehicle at the indicated bregma level are represented by an asterisk. Comparisons between groups were performed by Dunnets multiple comparison test *p<0.05, **p<0.01, ***p<0.001.

**Table S6. Cortex (Penumbra) Genes.**

|  | NPD1+RvD1- Avg Fold Change | Vehicle - Avg Fold Change | *n* Vehicle | *n* NPD1+RvD1 | test-statistic | pvalue |
| --- | --- | --- | --- | --- | --- | --- |
| Gpc4 | 20.669 | 1.196 | 8 | 8 | 60 | **0.001864802** |
| Cd163 | 123.505 | 1.344 | 8 | 8 | 59 | **0.002952603** |
| H2-D1 | 10.044 | 1.311 | 8 | 8 | 57 | **0.006993007** |
| Ptx3 | 21.499 | 1.662 | 8 | 8 | 56 | **0.01041181** |
| Cxcl10 | 18.946 | 1.691 | 8 | 8 | 55 | **0.014763015** |
| iigp1 | 11.615 | 1.107 | 8 | 8 | 55 | **0.014763015** |
| Ugt1a | 33.46 | 1.251 | 8 | 8 | 55 | **0.014763015** |
| C3 | 9.966 | 1.235 | 8 | 8 | 54 | **0.020668221** |
| Tm4sf1 | 24.215 | 1.188 | 8 | 8 | 54 | **0.020668221** |
| Amigo2 | 11.399 | 1.245 | 8 | 8 | 53 | **0.028127428** |
| Fcrls | 19.893 | 1.175 | 8 | 8 | 53 | **0.028127428** |
| Msr1 | 20.641 | 1.457 | 8 | 8 | 53 | **0.028127428** |
| Fizz1 | 94.017 | 3.233 | 5 | 6 | 27 | **0.03030303** |
| Tmem119 | 10.215 | 1.05 | 8 | 8 | 52 | **0.037917638** |
| Hmgb1 | 4.09 | 1.015 | 8 | 8 | 51 | **0.04988345** |
| Thbs 2 | 0.49 | 1.255 | 8 | 7 | 12 | 0.072105672 |
| Cd68 | 6.669 | 2.26 | 8 | 8 | 49 | 0.082983683 |
| Thbs 1 | 9.289 | 2.166 | 8 | 8 | 49 | 0.082983683 |
| Stat3 |  |  | 3 | 3 | 0 | 0.1 |
| Gbp2 | 2.962 | 1.123 | 8 | 8 | 48 | 0.104895105 |
| Osmr | 14.824 | 1.36 | 8 | 8 | 48 | 0.104895105 |
| Cd206 | 0.73 | 1.167 | 8 | 7 | 14 | 0.120590521 |
| C1qC | 2.419 | 1.05 | 8 | 8 | 47 | 0.13038073 |
| Cx3cr1 | 3.419 | 1.119 | 8 | 8 | 47 | 0.13038073 |
| Il1a | 0.92 | 1.948 | 8 | 8 | 17 | 0.13038073 |
| Nos2 | 4.325 | 2.134 | 8 | 7 | 41 | 0.151981352 |
| Cd40 | 1.33 | 1.363 | 8 | 7 | 16 | 0.189277389 |
| Aif1 | 1.346 | 1.111 | 8 | 8 | 19 | 0.194871795 |
| P2y12 | 4.488 | 1.202 | 8 | 8 | 45 | 0.194871795 |
| TGF beta | 1.74 | 1.569 | 8 | 8 | 19 | 0.194871795 |
| Tspo | 4.263 | 1.422 | 8 | 8 | 45 | 0.194871795 |
| Vim | 0.919 | 1.916 | 8 | 8 | 19 | 0.194871795 |
| Cd86 | 1.297 | 1.044 | 8 | 7 | 17 | 0.231857032 |
| Ggta1 | 0.803 | 1.223 | 8 | 7 | 17 | 0.231857032 |
| Cd14 | 7.084 | 1.741 | 8 | 8 | 44 | 0.234498834 |
| Cd45 | 1.459 | 1.733 | 8 | 8 | 20 | 0.234498834 |
| Il4 | 2.907 | 2.714 | 8 | 8 | 21 | 0.278632479 |
| Arg1 | 0.95 | 1.115 | 8 | 7 | 18 | 0.280963481 |
| Aspg | 2.927 | 1.521 | 8 | 7 | 18 | 0.280963481 |
| Fkbp5 | 0.461 | 1.086 | 5 | 5 | 7 | 0.30952381 |
| Gfap | 1.75 | 1.293 | 8 | 8 | 22 | 0.328205128 |
| Psmb8 | 0.882 | 1.323 | 8 | 8 | 22 | 0.328205128 |
| Emp1 | 1.567 | 1.415 | 8 | 7 | 19 | 0.335664336 |
| Sparcl1 | 0.669 | 1.127 | 6 | 8 | 16 | 0.344988345 |
| Igf1 | 0.905 | 1.18 | 8 | 8 | 23 | 0.382284382 |
| Il6 | 9.62 | 6.927 | 8 | 8 | 41 | 0.382284382 |
| Srgn | 0.793 | 1.309 | 8 | 8 | 23 | 0.382284382 |
| Cd32 | 3.095 | 2.657 | 6 | 6 | 24 | 0.393939394 |
| B3gnt5 | 1.24 | 1.587 | 8 | 7 | 20 | 0.396891997 |
| Cd11b | 4.38 | 1.757 | 8 | 7 | 20 | 0.396891997 |
| Dectin-1 | 4.112 | 4.378 | 8 | 7 | 20 | 0.396891997 |
| Ptgs2 | 5.047 | 2.436 | 8 | 7 | 20 | 0.396891997 |
| S100a10 | 2.769 | 1.201 | 8 | 8 | 40 | 0.441802642 |
| Timp1 | 3.188 | 5.439 | 8 | 8 | 24 | 0.441802642 |
| Sall1 | 2.736 | 1.394 | 8 | 7 | 21 | 0.463403263 |
| Cp | 1.125 | 1.816 | 8 | 8 | 25 | 0.505361305 |
| H2-T23 | 0.94 | 1.055 | 8 | 8 | 25 | 0.505361305 |
| Iba1 | 1.335 | 1.091 | 8 | 8 | 25 | 0.505361305 |
| Itgam | 1.874 | 2.733 | 8 | 7 | 34 | 0.535819736 |
| Lcn2 | 1.805 | 3.568 | 8 | 7 | 22 | 0.535819736 |
| Tnfa | 1.659 | 1.614 | 8 | 7 | 22 | 0.535819736 |
| Serping1 | 1.462 | 1.178 | 8 | 7 | 23 | 0.612587413 |
| C1qA | 1.125 | 1.115 | 8 | 8 | 37 | 0.645376845 |
| Il10 |  |  | 2 | 1 | 2 | 0.666666667 |
| beta2 integrin | 6.969 | 1.901 | 8 | 8 | 36 | 0.720901321 |
| Cd16 | 3.11 | 1.515 | 8 | 8 | 28 | 0.720901321 |
| Gpc6 | 2.234 | 1.185 | 8 | 8 | 28 | 0.720901321 |
| Slc10a6 | 1.057 | 1.119 | 8 | 8 | 28 | 0.720901321 |
| Steap4 | 2.118 | 1.54 | 8 | 8 | 36 | 0.720901321 |
| Aquaporin-4 | 0.891 | 1.226 | 8 | 8 | 29 | 0.798445998 |
| Hspb1 | 2.573 | 1.851 | 8 | 8 | 29 | 0.798445998 |
| INF gamma | 1.379 | 1.043 | 5 | 5 | 14 | 0.841269841 |
| Cd109 | 2.556 | 1.028 | 8 | 7 | 30 | 0.866511267 |
| IL1b | 3.456 | 2.902 | 8 | 8 | 34 | 0.878477078 |
| C1qB | 1.771 | 1.055 | 8 | 8 | 31 | 0.959129759 |
| S1pr3 | 2.456 | 1.372 | 8 | 8 | 33 | 0.959129759 |

Statistic results from Individual Wilcoxon-Mann-Whitney comparison of Saline vs NPD1+RvD1. *p<0.05, **p<0.01, ***p<0.001.

**Table S7. Normality Test for Gene Expression Experiment.**

| Astrocyte Genes | Vehicle | NPD1 +RvD1 |
| --- | --- | --- |
| Test for normal distribution |  |  |
| Kolmogorov-Smirnov test |  |  |
| KS distance | 0.08787 | 0.07496 |
| P value | >0.1000 | >0.1000 |
| Passed normality test (alpha=0.05)? | Yes | Yes |
| P value summary | ns | ns |
|  |  |  |
| Number of values | 45 | 45 |
|  |  |  |
| Microglia Genes | **Vehicle** | **NPD1 + RvD1** |
| Test for normal distribution |  |  |
| Kolmogorov-Smirnov test |  |  |
| KS distance | 0.1122 | 0.1031 |
| P value | >0.1000 | >0.1000 |
| Passed normality test (alpha=0.05)? | Yes | Yes |
| P value summary | ns | ns |
|  |  |  |
| Number of values | 31 | 31 |

Kolmogorov-Smirnov test for normality for gene expression experiments.

**Table S8. Normality Test for Bregma Distribution.**

|  | Saline | NPD1 (222µg/kg) | RvD1 (222µg/kg) | RvD1 + NPD1 3h | RvD1 + NPD1 4h | RvD1 + NPD1 5h | RvD1 + NPD1 6h |
| --- | --- | --- | --- | --- | --- | --- | --- |
| Test for normal distribution |  |  |  |  |  |  |  |
| Kolmogorov-Smirnov test |  |  |  |  |  |  |  |
| KS distance | 0.1544 | 0.2443 | 0.1564 | 0.2324 | 0.1352 | 0.2011 | 0.1682 |
| P value | >.100 | >.100 | >.100 | >.100 | >.100 | >.100 | >.100 |
| Passed normality test (alpha=0.05)? | Yes | Yes | Yes | Yes | Yes | Yes | Yes |
| P value summary | ns | ns | ns | ns | ns | ns | ns |
|  |  |  |  |  |  |  |  |
| Number of values | 9 | 9 | 9 | 9 | 9 | 9 | 9 |

Kolmogorov-Smirnov test for normality for Bregma level distribution data.

**Table S9. Normality Test for MRI Lesion Volume.**

| MRI Test for Normality |  |  |  |  |
| --- | --- | --- | --- | --- |
| Core MRI Lesion |  |  |  |  |
| Normality of Residuals |  |  |  |  |
| Test name | Statistics | P value | Passed normality test (alpha=0.05)? | P value summary |
| D'Agostino-Pearson omnibus (K2) | 4.73 | 0.094 | Yes | ns |
|  |  |  |  |  |
| Normality of Residuals |  |  |  |  |
| Test name | Statistics | P value | Passed normality test (alpha=0.05)? | P value summary |
| D'Agostino-Pearson omnibus (K2) | 2.566 | 0.2772 | Yes | ns |
|  |  |  |  |  |
| Normality of Residuals |  |  |  |  |
| Test name | Statistics | P value | Passed normality test (alpha=0.05)? | P value summary |
| D'Agostino-Pearson omnibus (K2) | 1.376 | 0.503 | Yes | ns |

Tests for normality for MRI lesion volume analysis data.
